# Surface Reconstructions in II–VI Quantum Dots

**DOI:** 10.1021/acsnano.3c09265

**Published:** 2024-01-03

**Authors:** Jordi Llusar, Indy du Fossé, Zeger Hens, Arjan Houtepen, Ivan Infante

**Affiliations:** † BCMaterials, Basque Center for Materials, Applications, and Nanostructures, UPV/EHU Science Park, Leioa 48940, Spain; ‡ Department of Chemical Engineering, Optoelectronic Materials, Delft University of Technology, Van der Maasweg 9, 2629 HZ Delft, The Netherlands; § Ikerbasque Basque Foundation for Science, Bilbao 48009, Spain; ∥ Physics and Chemistry of Nanostructures, Department of Chemistry, and Center of Nano and Biophotonics, Ghent University, B-9000 Gent, Belgium

**Keywords:** quantum dots, density functional theory, semiconductors, surface states, surface reconstructions

## Abstract

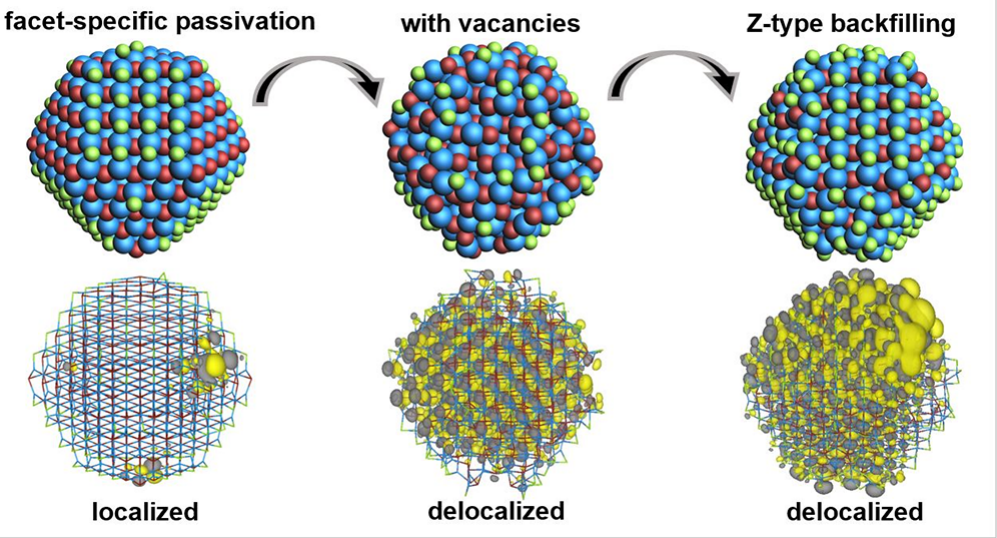

Although density functional theory (DFT) calculations have been crucial in our understanding of colloidal quantum dots (QDs), simulations are commonly carried out on QD models that are significantly smaller than those generally found experimentally. While smaller models allow for efficient study of local surface configurations, increasing the size of the QD model will increase the size or number of facets, which can in turn influence the energetics and characteristics of trap formation. Moreover, core–shell structures can only be studied with QD models that are large enough to accommodate the different layers with the correct thickness. Here, we use DFT calculations to study the electronic properties of QDs as a function of size, up to a diameter of ∼4.5 nm. We show that increasing the size of QD models traditionally used in DFT studies leads to a disappearance of the band gap and localization of the HOMO and LUMO levels on facet-specific regions of the QD surface. We attribute this to the lateral coupling of surface orbitals and the formation of surface bands. The introduction of surface vacancies and their a posteriori refilling with Z-type ligands leads to surface reconstructions that widen the band gap and delocalize both the HOMO and LUMO. These results show that the surface geometry of the facets plays a pivotal role in defining the electronic properties of the QD.

## Introduction

Computational studies have played a crucial role in the advancement of our understanding of colloidal quantum dots (QDs) over the past two decades.^1−4^ Density functional theory (DFT) calculations have allowed the investigation of QD models with realistic structures and ligand-terminated surfaces that closely resemble experimental observations.^1,5^ However, despite significant improvements in computational power, most DFT studies still rely on model systems that are considerably smaller than real QDs found in experiments.^1,6−8^ For example, excluding ligands, a spherical CdSe QD with a diameter of 5 nm contains approximately 2400 atoms, while DFT models typically consist of fewer than 500 atoms (∼3 nm diameter).^9−14^ Although smaller models are suitable for studying local surface configurations,^13−15^ they may overlook surface localized states that emerge when the size of a QD, and thus of its facets, is expanding. Hence, these models likely do not provide an accurate representation of larger QDs. Moreover, for a precise description of core/shell(/shell) particles, QD models are needed that represent core and shell(s) with the correct thickness, which would make these models nearly identical in size to their experimental counterparts.

Previous investigations of QD systems with diameters of >5 nm have employed various computational methods. Tight-binding calculations have been effective for accurately reproducing experimentally observed absorption spectra.^16−19^ Pseudopotential methods are useful for studying carrier dynamics and the electronic structure of QDs.^20−23^ However, both methods are less suitable to predict the geometry of the QD surface.^5^ Furthermore, these calculations often employ pseudohydrogen passivation,^17,18,20,21,24^ which does not resemble the experimentally present carboxylate or amine ligands and, as such, is not appropriate for a chemical description of the QD surface.^5^ The pseudohydrogen passivation turns all surface atoms of a QD into four-coordinated surface species, a situation that neglects the possibility that the abrupt termination of a QD by different facets yields undercoordinated surface atoms.^13,14,25,26^ Recent developments in classical force fields tailored specifically to QDs have enabled the study of large QDs with realistic ligand capping at a fraction of the computational cost.^27,28^ However, these force field methods cannot predict the electronic structure of the QD, and thus the formation of localized states through, for example, electron charging and discharging. For detailed analyses of surface states and their impact on the electronic properties of QDs, DFT remains the most effective choice.^5^

Nevertheless, the literature on DFT studies of QD models containing over 500 atoms is severely limited. Some reports employ pseudohydrogen passivation, similar to pseudopotential and tight-binding studies, but this approach fails to accurately describe the chemical nature of the QD surface.^29,30^ In 2014, Voznyy and Sargent presented a CdSe QD model comprising approximately 1200 atoms, excluding the ligands, that was passivated with chloride ions. However, the HOMO and LUMO of this QD were localized on surface facets and separated by a negligible HOMO–LUMO gap.^31^ A similar surface localization of the HOMO resulted from DFT calculations on CdSe nanoplatelets.^32^ These findings are inconsistent with the well-established particle-in-a-box model of QDs,^33,34^ which predicts delocalized orbitals, as experimentally observed with scanning tunneling microscopy,^24,35,36^ and size-tunable band gap of QDs.^37,38^

Surface localized orbitals with energy inside the bulk band gap may result from the reduced coupling of atomic orbitals to surface atoms. For flat semiconductor surfaces in vacuum, the formation of facet specific 2D surface bands is well-known and is often countered by the spontaneous formation of surface vacancies. To address this facet truncation problem, Voznyy and Sargent have indeed introduced surface vacancies, leading to a slightly n-doped QD with delocalized states.^31^ Nonetheless, additional studies are needed to explore the impact of surface vacancies and other factors on the QD electronic properties.

In the present study, we show that larger Cd- and Zn-chalcogenide model QDs that are terminated by low-index bulk facets feature increasingly surface localized HOMO and LUMO states with a negligible HOMO–LUMO gap. We demonstrate that this deviation from experimental findings can be addressed by introducing reconstructed surfaces that feature surface vacancies analogous to those observed on flat semiconductor surfaces. Additionally, we explore refilling these vacancies with Z-type ligands and find that it leads to similar delocalization of the HOMO–LUMO levels. This enables us to put forward a QD model without vacancies that retains a high ligand concentration as observed for experimental QDs and displays delocalized HOMO and LUMO levels separated by a sizable HOMO–LUMO gap, in line with experimental findings. Finally, we demonstrate that this approach can be used to build models for so-called type-I and type-II core–shell QDs that avoid, again, surface localization of HOMO and LUMO.

## Results and Discussion

### Issue with Large QD Models

**Figure 1:**
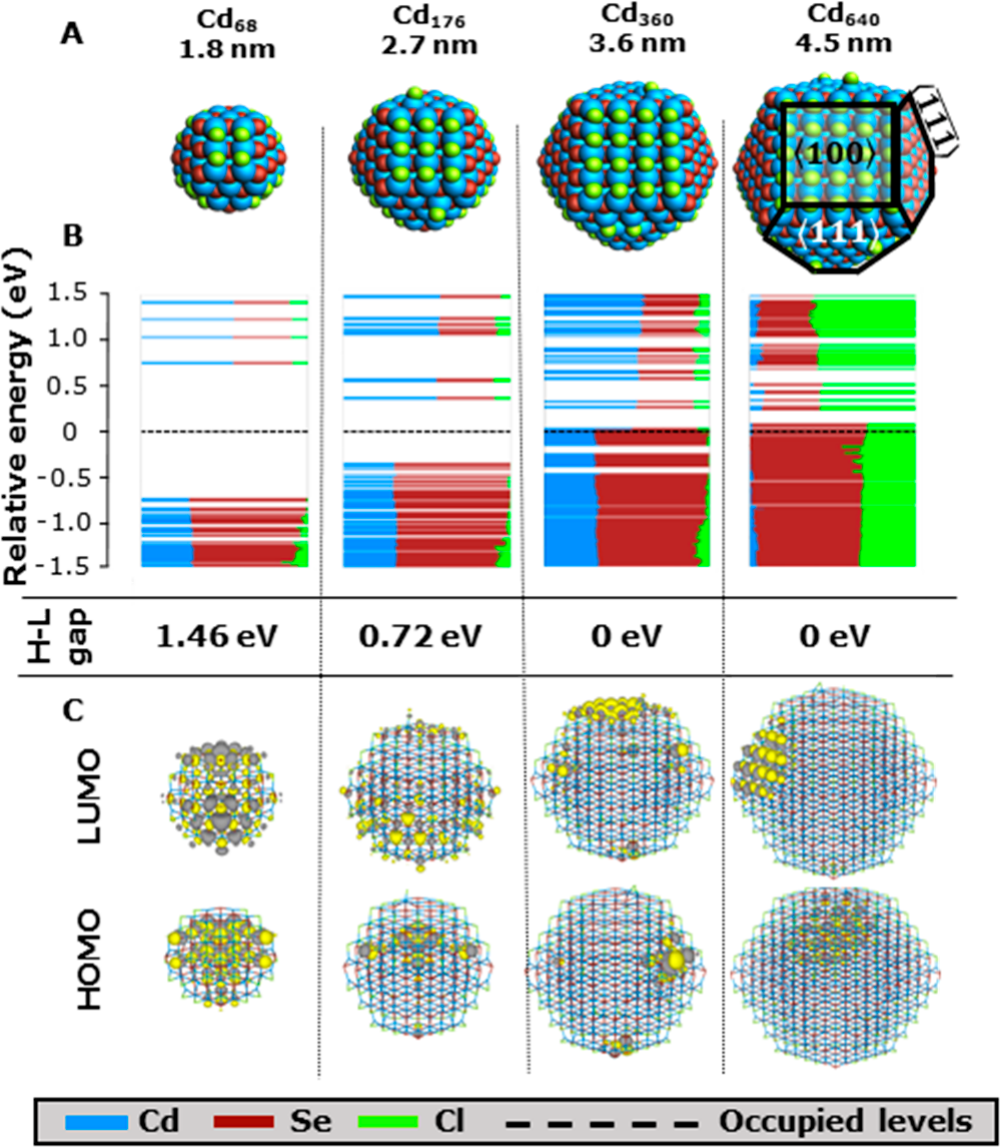
Effect of increasing the size of the QD model. (A) Structure of CdSe QD models with compositions of Cd_68_Se_55_Cl_26_, Cd_176_Se_147_Cl_58_, Cd_360_Se_309_Cl_102_, and Cd_640_Se_561_Cl_158_, respectively. (B) DOS for each QD with their respective HOMO and LUMO gap energies. Every horizontal line corresponds to an MO. The length of the colored line segments indicates the contribution of each element to an MO. Levels below the dotted line are filled with two electrons; levels above the line are empty. (C) Contour plots of the HOMO and LUMO of each system.

The zinc blende Cd_68_Se_55_Cl_26_ model system, which is illustrated in the leftmost part of Figure 1A, served as a reference structure for multiple previous studies.^13,15,43−48^ This model system, henceforth termed Cd68, is roughly spherical, cation-rich, and charge balanced. Charge balance is achieved through passivation with chloride ligands in lieu of the experimentally often-employed oleate ligands. Ligand distribution on the facets is primarily based on a previous work by Cosseddu and co-workers.^45^ The diameter of this QD model is approximately 1.8 nm, as measured from one corner of a ⟨111⟩-facet to the furthest corner of the ⟨111⟩-facet on the opposite side; a size significantly smaller than a typical experimental CdSe QD. The density of states (DOS), as well as the shapes of the HOMO and LUMO, are shown in Figure 1B,C, respectively. For the Cd_68_ model, both the HOMO and the LUMO are delocalized over the entire QD and separated by a HOMO–LUMO gap of 1.5 eV. Further details of this system may be found elsewhere.^13^

In line with the prediction of a particle-in-a-box model, increasing the QD diameter to 2.7 nm (Cd_176_) causes a decrease in the HOMO–LUMO gap. However, Figure 1C shows that the HOMO of this model QD no longer uniformly extends across the entire QD. Further increasing the QD size to 3.6 (Cd_360_) and 4.5 nm (Cd_640_) results in clearly surface localized HOMO and LUMO states—specifically on the Se-terminated - and Cd-terminated ⟨111⟩-facets, respectively—separated by a negligible HOMO–LUMO gap.

A versatile tool commonly used for quantifying the degree of localization of the wave function is the inverse participation ratio (IPR), which ranges from 1/*N*_atoms_ for complete delocalization to 1 for complete localization, where *N*_atoms_ are the number of atoms in the system. To compare systems of various sizes, it is more convenient to use a normalized participation ratio (PR), defined below, which ensures a value of 0% (for complete localization) and 100% (for complete delocalization) regardless of the number of Cd and Se atoms in the system:
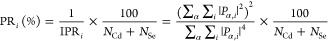
Here, (I)PR_*i*_ (%) is the (inverse) fractional participation ratio of the *i*th molecular orbital (MO)_*i*_, *P*_*α,i*_ is the weight of the (MO)_*i*_ on atom α, and *N*_Cd_ and *N*_Se_ are the numbers of Cd and Se atoms in the inorganic QD core, respectively. Note that the number of ligand atoms is specifically not included, since their contribution near the band edges is generally small.

**Figure 2:**
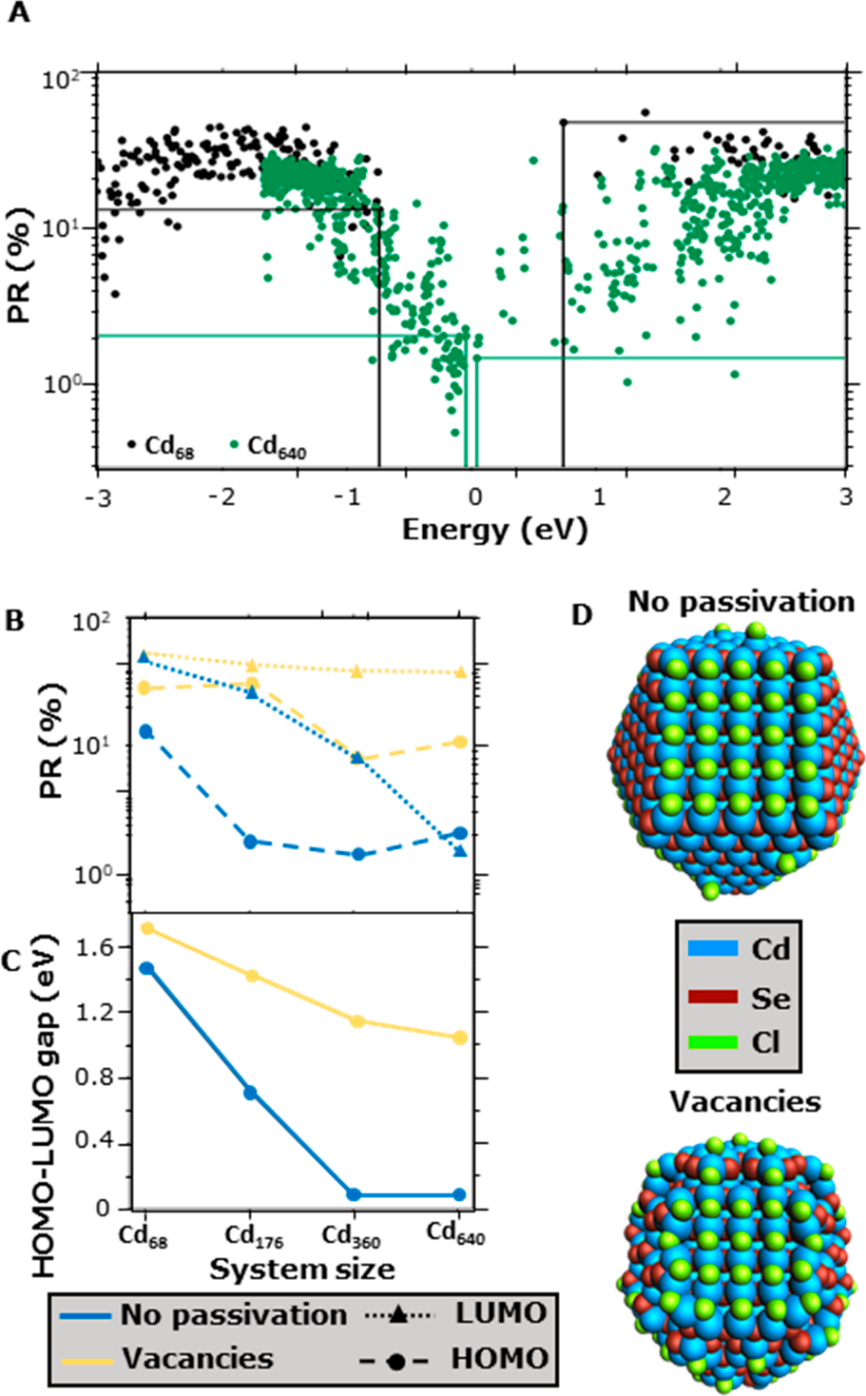
Left panel: Effect of surface reconstruction. (A) PR of Cd68 and Cd640. Each data point corresponds to a MO. The horizontal and vertical lines indicate the PR and energy of the HOMO and LUMO of both systems. (B) PRs of the HOMO (filled circles and dashed lines) and LUMO (filled triangles and dotted lines) as the size of the QD increases. (C) HOMO–LUMO gap of different sizes of QDs. The blue lines stand for nonpassivated QDs, whereas the yellow lines represent QDs with surface vacancies. (D) Structures of Cd_640_ with no passivation and surface vacancies.

Figure 2A shows that the HOMO and LUMO of Cd_68_ are delocalized over ∼10–60% of the atoms in the model QD. The HOMO and LUMO levels, represented by the black horizontal lines, exhibit similar participation ratios, indicating no significant localization for this QD size. For Cd_640_, shown in green in Figure 2A, the PRs of the levels deep inside the VB and CB are similar to those of Cd_68_. However, the PRs decrease significantly near the HOMO and LUMO, which reflects the localization of these states at the QD surface; see Figure 1C. Figure 2B displays the PRs of the HOMO and LUMO for all four QD sizes, where especially the PRs of the HOMO drop from ∼13% for Cd_68_ to ∼1–2% for the three largest QDs.

Likewise, the PRs of LUMO monotonically decrease from ∼47 to 1.5%. Figure 2C shows that the HOMO–LUMO gap concomitantly decreases with increasing QD size, reaching a negligibly small value of 0.08 eV for Cd_360_ and Cd_640_, which would render such QDs metallic. These results confirm and quantify the conclusion that increasing the size of the QD leads to surface localization of the band edges; see Figure 1C and, for further discussion, Section S2 in the Supporting Information (SI).

The localization of HOMO and LUMO on the QD surface and the disappearance of the HOMO–LUMO gap do not agree with multiple experimental findings. The possibility of attaining near-unity photoluminescence quantum yield (PLQY),^49,50^ the size-tuneability of the band gap,^37,38^ and the delocalized orbitals measured by scanning tunneling microscopy^24,35,36^ are consistent with the traditional description of the band edges through particle-in-a-box models, which predict fully delocalized HOMO and LUMO states and a HOMO–LUMO gap larger than the bulk band gap.^33,34^ Therefore, we assume that these computational results represent a situation that is not found in real QDs. This can have two possible causes: (1) the computational method is flawed, or (2) the atomistic structure of the modeled QD is wrong.

In the first case, we know from previous works that the PBE functional underestimates the band gap;^51^ however, it still predicts a band gap of approximately 0.5 eV for bulk CdSe, which should therefore be the limit for large QDs. Additionally, as described in more detail in the SI, the choice of the basis set or the exchange–correlation functional does not qualitatively affect the results. We conclude that the computational method is not at fault here. In the second case, the atomistic structure of the model QD might fail to reproduce the key characteristics of the QDs observed in experiments. One possible explanation is that ligands play a pivotal role in modulating the band gap of QDs. In experiment, QDs are often completely covered with X-type oleate ligands, L-type ligands like amines or phosphines, and Z-type ligands like CdCl_2_ or Cd(RCOO)_2_. Previous studies have shown that the addition of L-type ligands or the replacement of chloride X-type ligands with thiolates has little effect on the electronic structure of small Cd_68_ models.^13^ Interestingly, as illustrated in Figure S2, these results hold true for the large Cd_360_ model, aligning with the earlier conclusion for Cd_68_. The effect of adding Z-type ligands will be discussed below.

Another potential distinction between the modeled and real structures may lie in the intrinsic instability of the QD facets derived from cutting the QD out of the bulk material. This instability resembles that observed in flat semiconductor surfaces,^43^ where localized levels within the band gap might emerge at facets, edges, and corners of a finite crystal. In the SI, we provide an in-depth exploration of the formation of intragap localized states at the edges of 1D diatomic chains and beyond, based on a tight-binding approach developed by Harrison.^52^ In essence, within a 1D diatomic chain and under a regime of weak coupling between neighboring orbitals, the uppermost valence band state would be a purely antibonding combination of Cd and Se s orbitals, while the lowermost conduction band state a fully bonding combination of Cd and Se p orbitals; see Figure S5. In contrast, as the coupling between adjacent atomic orbitals increases, these two bands invert (band inversion). As illustrated in Figure S6, under such conditions, two surface states emerge between band-edge states: one localized on the anion at the chain’s left edge and the other on the cation at its right edge. This logic can be extended to 2D, where intragap localized states manifest at the edge of the 2D lattice (Figure S7) and to 3D diatomic crystals. In the latter, following the same rationale, the localized state becomes facet-specific. Therefore, we can infer that, in binary AB crystals with an s-like antibonding CB minimum and p-like bonding VB maximum, like CdSe or InP, the presence of surface localized states in finite 3D crystals is a common occurrence and should be recognized as an inherent feature rather than a flaw in the computational methodology. However, under these conditions, these facets can still become intrinsically unstable and can reconstruct under experimental conditions. Such surface reconstructions are discussed next.

### Surface Reconstructions

**Figure 3:**
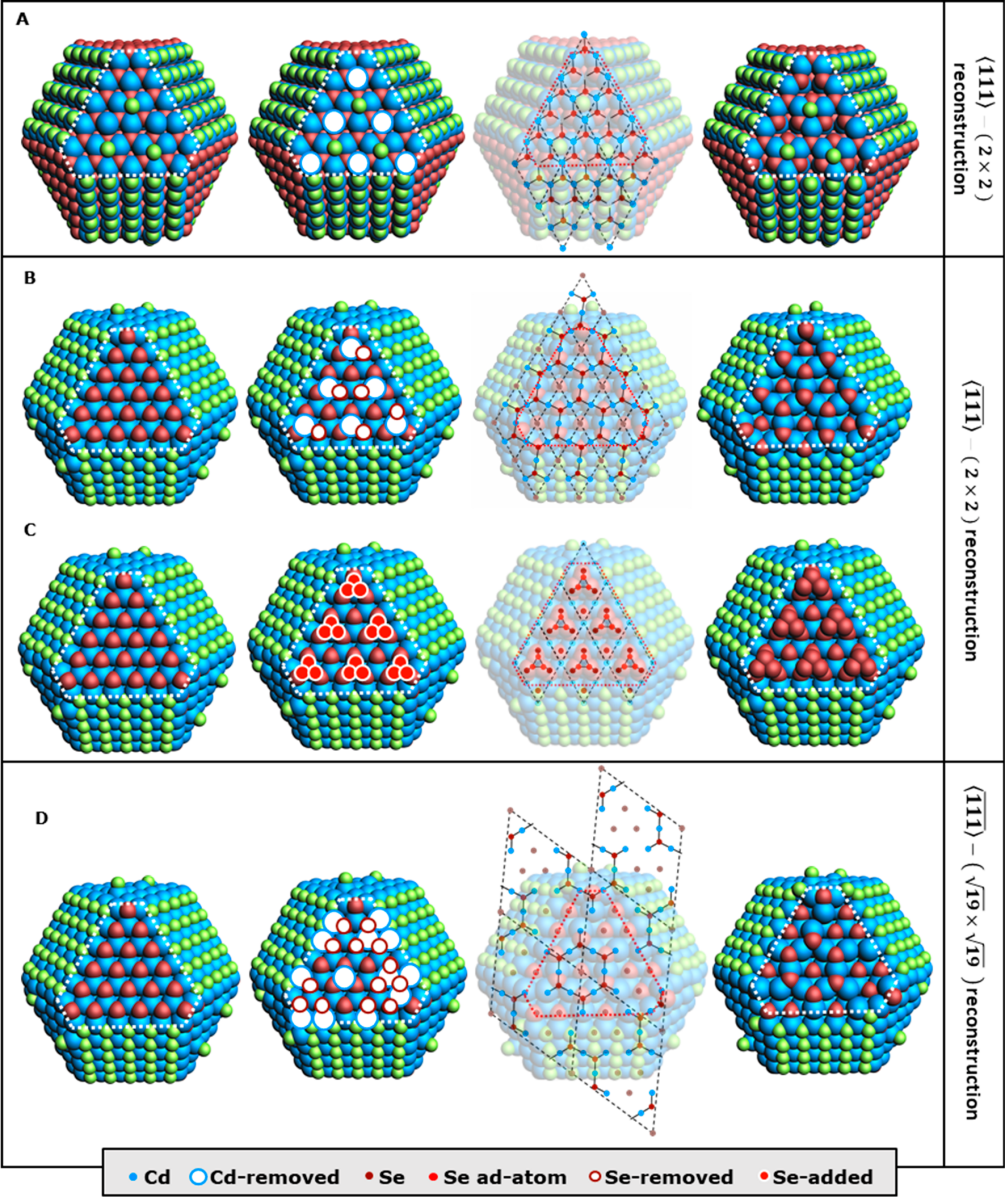
Possible reconstructions of ⟨111⟩- and -facets in the Cd_640_ model. (A) Reconstruction of the ⟨111⟩-facet based on a cation vacancy. (B) Reconstruction of the -facet as proposed by Chadi.^60^ (C) and (D) reconstructions of the -facet as observed by Biegelsen et al.^61^ The columns depict the unreconstructed model, the addition or removal of atoms (white circles), the pattern for adding or removing atoms, and the final reconstructed model. Note that the structures, for depiction purposes, are not optimized since this can lead to small distortions of the reconstructions near the edge or corner sites. White and red dotted lines indicate the treated facets.

It is well-known from studies on the surfaces of bulk semiconductors that facets obtained by cutting the bulk are not always stable.^43^ As indicated in Figure 1A, our model QDs exhibit ⟨100⟩-, ⟨111⟩-, and -facets, which all have been shown to reconstruct for flat surfaces in vacuum.^53^ Although much of the previous research on surface reconstructions of compound semiconductors has been conducted on GaAs surfaces, the findings are expected to be applicable to II–VI compounds.^53^ In general, the ⟨100⟩-facets contain a significant amount of dicoordinated Cd ions. However, the introduction of X-type ligands effectively passivates these ions, yielding bulklike 4-fold coordination. In contrast, the ⟨111⟩- and -facets exhibit a higher prevalence of tricoordinated Cd and Se, respectively. In the model systems we employ, these facets are not as effectively passivated by ligands compared to the ⟨100⟩-facets, and they can undergo a reconstruction commonly observed in GaAs ⟨111⟩ surfaces, as shown in Figure 3A, where 25% of the surface cations are replaced by a vacancy.^53−55^ This particular reconstruction has also been predicted for ZnSe,^56^ and similar results have been obtained for wurtzite CdSe.^57^ For the  surface, multiple structures have been proposed, as will be discussed below.

### Formation of Superficial Cation Vacancies

We explored various possibilities for incorporating surface reconstructions into our QD models. For the ⟨111⟩-facets, the cation-vacancy reconstruction depicted in Figure 3A stands out as the most favorable option. This specific reconstruction presents the sole pattern mentioned in the experimental literature for zinc blende materials.^54,58,59^ In contrast, for the -facets, three surface reconstructions have been reported that could fit on the relatively small facets of the Cd_640_ model: (1) the first is based on the work by Chadi,^60^ who proposed a multivacancy model in which both cation and anion vacancies lead to the ring structure displayed in Figure 3B. This reconstruction features a suitably small unit cell that can be accommodated within a typical QD facet. It is obtained by simultaneously removing one Cd and one Se atom to preserve the overall charge balance of the system without requiring the addition or removal of Cl ions at other facets. (2) The second reconstruction is based on the work by Biegelsen et al.,^61^ who utilized scanning tunneling microscopy to demonstrate the formation of a trimer of anion-adatoms forms on the GaAs  surface as depicted in Figure 3C.

We have however discarded this trimer structure due to charge balance concerns: to introduce numerous anions onto the -facets, it would be necessary to remove chloride ligands from the ⟨100⟩-facets, which, in turn, would significantly destabilize these facets.^43^ (3) The final reconstruction——is displayed in Figure 3D and could, as stated in ref (60) for GaAs, be experimentally obtained by annealing the reconstructed structure——in Figure 3B.^61^ Note that the structure of Figure 3B can be obtained from Figure 3D by shifting the hexagonal structures of the latter so that the protruding cations are turned into bridging atoms.^60,61^ The structure in Figure 3D however presents challenges due to its large unit cell size, making it difficult to fit onto a QD facet. Moreover, due to the higher cation/anion ratio compared to the structure in Figure 3B, additional chloride ligands need to be placed on the ⟨111⟩-facets. As demonstrated in Figure S8 for Cd_640_, this leads to the formation of undesired localized states. Taking into account all of these factors, we have opted to proceed with option 1 on the -facets, i.e., the reconstruction shown in Figure 3B.

The introduction of surface reconstructions, as depicted in Figure 2C, leads to an increase in the HOMO–LUMO gap for all QD sizes. The effect is particularly pronounced for Cd_360_ and Cd_640_, with the band gap transitioning from a metallic nature (∼0 eV) to values exceeding 1.0 eV. Figure 2B demonstrates similar improvements in the delocalization of HOMO and LUMO orbitals. Regardless of the QD size, the LUMO exhibits delocalization of approximately 40%. Note that, due to the introduction of vacancies, the number of Cd atoms is slightly lower than that indicated by the name (i.e., 68, 176, 360, and 640), the total compositions being Cd_56_Se_43_Cl_26_, Cd_152_Se_123_Cl_58_, Cd_324_Se_281_Cl_86_, and Cd_592_Se_517_Cl_150_, respectively. To explain the reversal of the HOMO–LUMO gap closure, one can refer to the autocompensation effect observed on bulk surfaces. This effect arises from the formation of an equal number of cation- and anion-dangling bonds on their respective facets, prompting a spontaneous rearrangement of surface atoms to eliminate the metallic surface band. This process resembles a Peierls transition.^53^

### Backfilling of the Surface Vacancies with Z-Type Ligands

**Figure 4:**
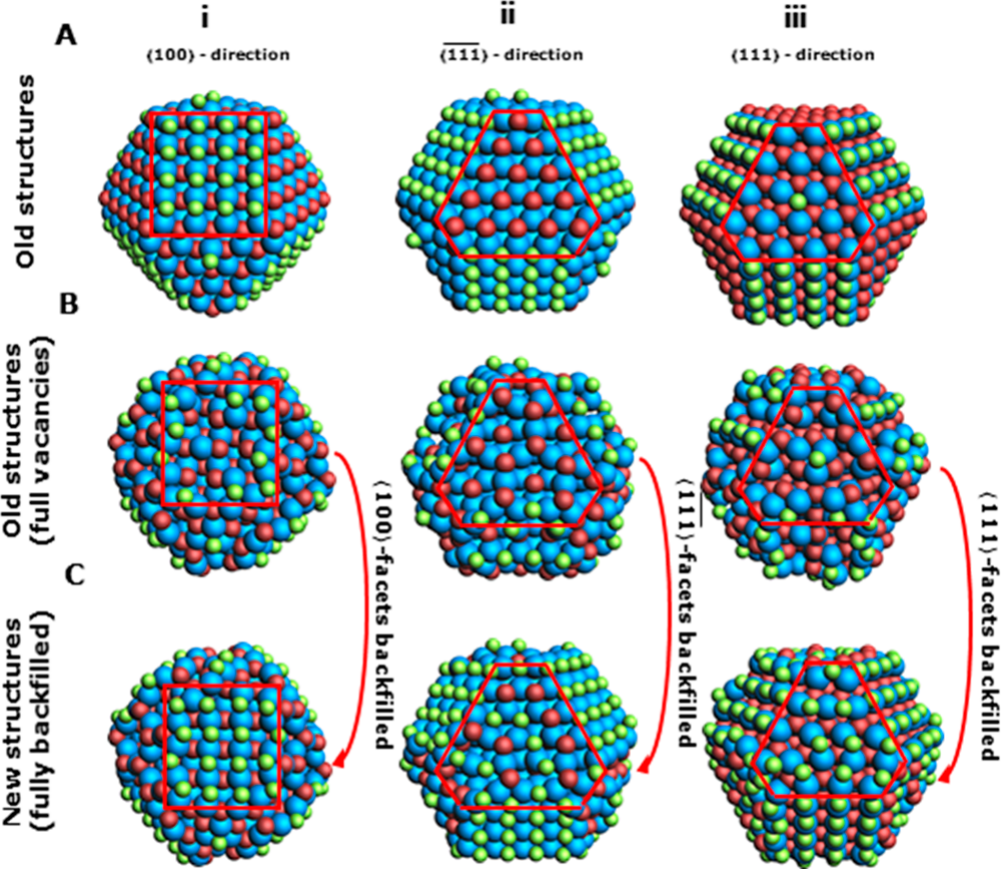
Comparison among QD structures along different directions. (A) Sketch of a pure (without vacancies) QD, (B) full-vacancies QD, and (C) completely backfilled QD. Red lines provide a guide-to-eye to delimit the correspondent facets. Unlike panel C, both panels A and B are referred to as old structures since they are not considered the definitive structures.

While the model with vacancies has successfully addressed the problem of preventing the formation of localized states on QD facets, the QD model with vacancies on the  and ⟨111⟩ features a ligand surface concentration of only 2.8 nm^–2^, which is below the 3.5 nm^–2^ typically reported in experiments.^62^ Therefore, we considered that this model does not represent the actual surface composition of the experimental QDs and decided to refill the previously introduced vacancies by adding a CdCl_2_ Z-type ligand to each vacant site. This backfilling process provides a quite uniform distribution of Cl ligands, unlike the traditional QD model illustrated in Figure 4A, where the ligands are placed largely on the ⟨100⟩ surfaces.

To perform the backfilling on the Cd_360_ QD model and obtain a uniform distribution of ligands, we decided to make our approach as general as possible. Although not needed to obtain delocalized HOMO and LUMO levels, we have additionally created vacancies on the ⟨100⟩-facets, using the pattern shown in Figure S9, to explore a wider range of surface modifications. Therefore, the geometry depicted in Figure 4B(i–iii), which has an initial ligand density of 1.8 ligands/nm^2^, is our starting point for increasing the CdCl_2_ surface coverage by refilling one by one all of the vacancies with CdCl_2_ molecules following a specific order: ⟨100⟩, , and ⟨111⟩, as shown in Figure 4C(i–iii), respectively. This order is not arbitrary, because we expect that the dicoordinated Cd atoms on the ⟨100⟩-facets make this facet more reactive. Consequently, it becomes filled first.^63−65^ On the other hand, the - and ⟨111⟩-facets are expected to exhibit similar ligand stabilization, although it remains unclear which of the two facets is more stable. To assess the stability of each facet, we computed the complexation energy, Δ*E*_comp_,
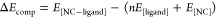
where *n* represents the number of ligand monomers in vacuum, *E*_[NC–ligand]_ is the energy of the complexed-nanocrystal energy, *E*_[ligand]_ is the energy of the ligand monomers in vacuum, and *E*_[NC]_ is the energy of the noncomplexed nanocrystal.

We obtained average complexation energies of −50.5, −32.1, and −22.6 kcal/mol for the ⟨100⟩-, -, and ⟨111⟩-facets, respectively. To do so, we considered as the initial system the one depicted in Figure 4B(i) and gradually added CdCl_2_ on each facet. Interestingly, after implementing this backfilling procedure, the HOMO–LUMO gap energy remains wide open and both the HOMO and the LUMO continue to exhibit delocalization consistently, even when all vacant sites are filled.

This result stems from the uniform rearrangement of the CdCl_2_ molecules across the whole QD, especially those placed on - and ⟨111⟩-facets, allowing both the Cd and Se to maintain a high coordination number larger than or equal to 3. The final QD model presents a high ligand density at approximately 5 ligands/nm^2^, which is larger than the experimentally observed value of 3.5 ligands/nm^2^.^62^ This indicates that to prepare a QD model for calculations that match the experimental ligand density, it is important to follow the order of complexation energies. To achieve a ligand density of 3.5 ligands/nm^2^, one should first completely fill the ⟨100⟩-facets, followed by 45% filling of the -facets. The ⟨111⟩-facets remain ligand free in this case. We note that, since the procedure starts from the vacancy-rich structures depicted in Figure 4B, the ⟨111⟩-facets remain full of vacancies and the -facets contain 55% of the vacancies. Once this experimental ligand density is achieved, the theoretical Cd:Se ratio yields 1.19. This value falls within the range of Cd/Se ratios reported in the studies by Karel Čapek et al.^66^ and Fritzinger et al.,^67^ which are 1.16 and 1.23, respectively. This procedure indicates that for ligand passivated surfaces, stable solutions with considerable HOMO–LUMO gap energies can be obtained without introduction of vacancies. The effects of vacancies and ligand passivation are similar in the sense that both prevent the formation of surface bands.

### Transferability to Other QD Models

**Figure 5:**
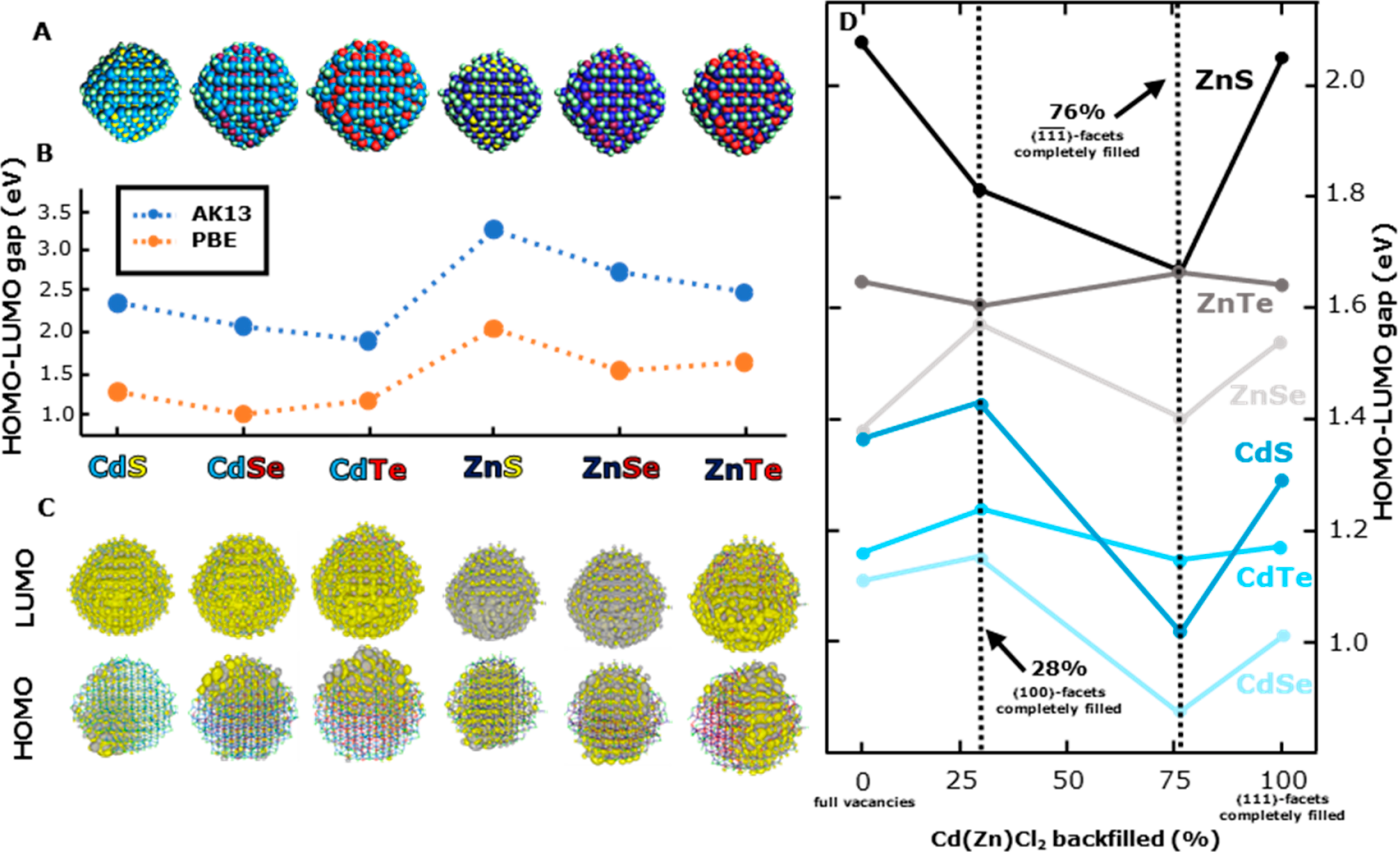
(A) Sketch of the different Cd(Zn)_360_ models used. (B) Evolution of the HOMO–LUMO gap energy as a function of the composition of the QD. The colors displayed on the names of the materials represent the atom colors in part A. Notice the comparison between functionals PBE (orange) and AK13 (blue). (C) Contour plots of the HOMO and LUMO of each system. (D) HOMO–LUMO gap energy tuning based on the percentage of Cd(Zn)Cl_2_ monomers backfilled in the different QDs. Blue and black-gray tonality lines represent QDs that are comprised of Cd and Zn cations, respectively.

To test the generality of these results, we extended the approach developed for the Cd_360_ model to different II–VI systems: CdS, CdTe, ZnSe, ZnS, and ZnTe for the case of maximum surface coverage. Interestingly, we did not observe any significant differences; indeed, similarly to the Cd_360_ structure, all of these QD models featured delocalized HOMO and LUMO states and also wide HOMO–LUMO gap energies; see Figure 5. Note that the HOMO–LUMO gap energies obtained in this work show the same trend among II–VI structures with relation to the bulk band gap energy when using PBE functionals.^68^ This implies that the formation of surface localized states, and their elimination by appropriate passivation with Z-type ligands, is a general feature of II–VI zinc blende QDs and, in turn, a general feature of II–VI and III–V.^13^ We have also tested the recently developed AK13 functional that should provide a better estimate of the band gaps and do indeed find that the band gaps follow the expected trend for different semiconductors and are roughly in line with the band gaps expected for these QD sizes.^68^

**Figure 6:**
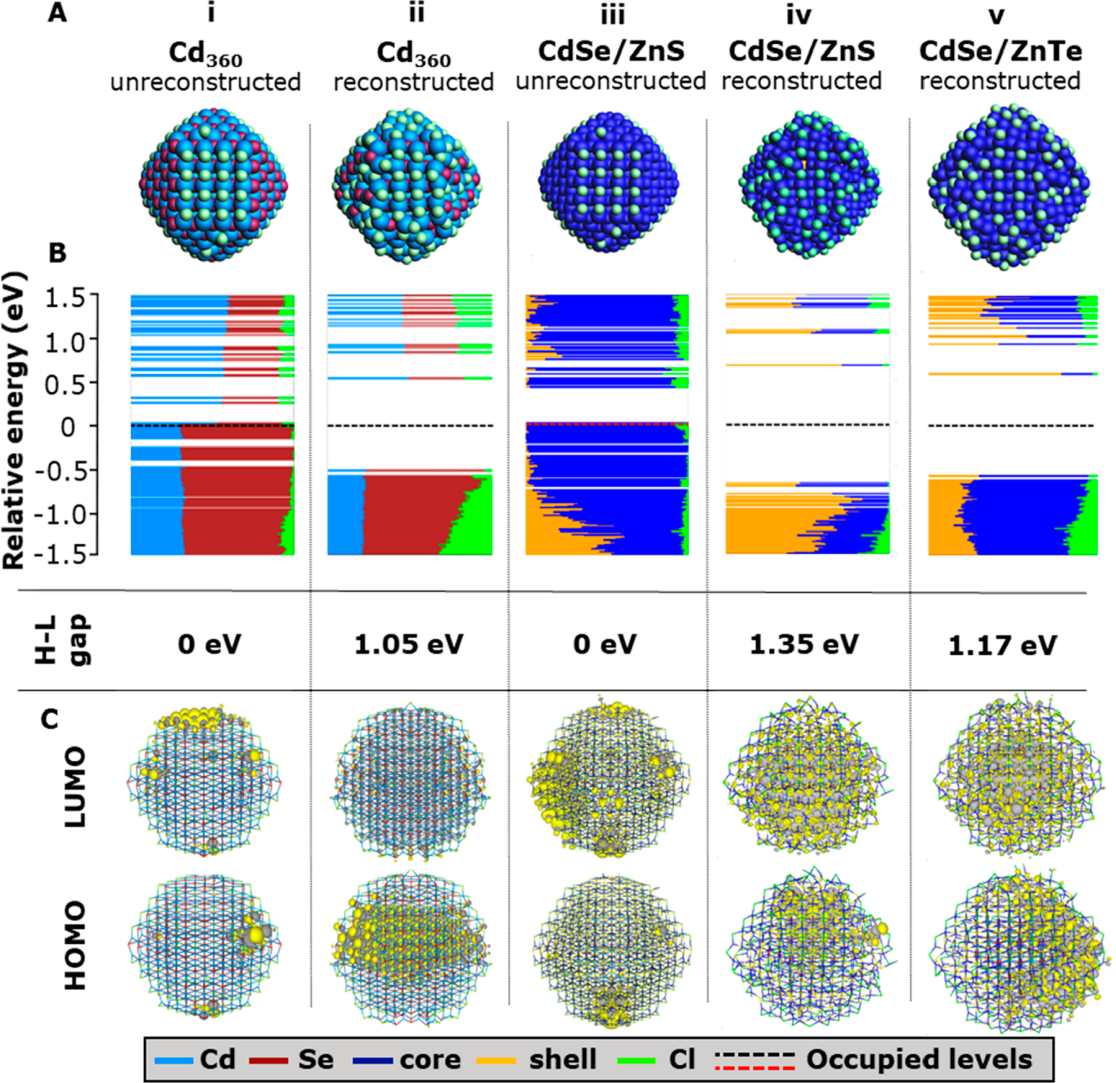
Comparison of the Cd_360_ model with the CdSe/ZnS core/shell system with and without surface reconstruction. Only the reconstructed versions of CdSe/ZnS and CdSe/ZnTe core/shell models are compared one to each other. (A) Structures of both the unreconstructed and reconstructed core-only and the CdSe/ZnS core/shell models and reconstructed CdSe/ZnTe core/shell model. (B) DOS of each system with their respective HOMO–LUMO gap energies. (C) Contour plots of the HOMO and LUMO of each system.

As reconstruction of the QD surface makes it possible to create larger QD models that still show the expected behavior (i.e., a band gap and delocalized band edges), the fully reconstructed model of Cd_360_ can now also be used to create archetypal core/shell structures, such as type-I and type-II heterostructures. First, in Figure 6, we compare the Cd_360_ model with a CdSe/ZnS core/shell QD, both without and with surface reconstruction. The core/shell model consists of a Cd_68_Se_55_ core (that is, the smallest QD model in Figure 1) and ∼2 layers of ZnS shell, leading to a total composition of Cd_68_Se_55_/Zn_292_S_254_Cl_102_ and Cd_68_Se_55_/Zn_292_S_226_Cl_158_ for the unreconstructed and reconstructed models, respectively (see Figure S10 for a view of the inside of the reconstructed core/shell QD). The unreconstructed CdSe/ZnS model in Figure 6A(iii) clearly shows surface localized HOMO and LUMO wave functions and a metallic DOS, very similar to the pure CdSe model of the same size in Figure 6A(i). This shows that the appearance of a band of surface localized states is a property of the surface facet and is not sensitive to the core. The reconstructed CdSe/ZnS system shown in Figure 6A(iv) exhibits significantly delocalized HOMO and LUMO wave functions and a well-defined HOMO–LUMO gap.

We notice that, in the reconstructed CdSe/ZnS system, the HOMO is overall mainly localized on the CdSe core with some leakage to the ZnS shell, while the LUMOs are mainly in the shell. This can be seen in the HOMO and LUMO wave function plots in Figure 6C and more clearly in the relative contributions of core (orange) and shell (dark blue) to the HOMO and LUMO levels in the density-of-states plot in Figure 6B(iv).

Employing a typical band-alignment classification, this suggests a quasi-type-I band alignment against the expected pure type-I. Second, contrary to the CdSe/ZnS system, the CdSe/ZnTe core/shell QD, with composition Cd_68_Se_55_/Zn_292_Te_226_Cl_158_, displays features of a type-II alignment in agreement to that observed in the bulk, e.g., the VB edge delocalized in the shell and CB mostly defined in the core; see Figure 6C. In QDs quantum confinement of the core and shell can influence the relative alignment of the two materials. Furthermore, the band alignment will be sensitive to the charge distribution on the surface and in the ligand shell, which in the calculations could still deviate from the experimental surface composition. This may explain why in these relatively small (but computationally large) core–shell models, the observed alignment differs from what is expected in the bulk. Therefore, for further study of these core/shell models, the effect of the core and shell size and surface composition must be carefully considered. However, these results show that given proper termination of the QD surface DFT can be used to study larger QDs and core/shell systems.

## Conclusions

In conclusion, we used DFT calculations to study the electronic properties of larger QDs (up to ∼4.5 nm in diameter) with realistic surface passivation. It is found that increasing the size of traditional QD models leads to the disappearance of the band gap and the localization of HOMO and LUMO on the surface. We have shown that the introduction of surface reconstructions on all of the ⟨100⟩-, -, and ⟨111⟩-facets gives rise to the delocalization of both the HOMO and LUMO and a significant increase in the band gap. Furthermore, refilling these surface reconstructions with Z-type ligands resulted in a similar delocalization of the HOMO and LUMO levels along with the appearance of band gap energies closely aligned with the experimental values. These results show that the QD surface plays a pivotal role in the delocalization of the wave function, which depends not only on the presence of (in)organic ligands but also on the surface geometry of the QD facets. The new surface geometries identified in this work enable the study of quantum dots of realistic sizes as well as technologically important core–shell QDs with density functional theory methods.

## Methods

Geometry optimizations were performed at the DFT level using a Perdew–Burke–Ernzerhof exchange–correlation functional (PBE)^39^ and double-ζ basis set, as implemented in the CP2K quantum chemistry software package.^40^ Relativistic effects were considered through the effective core potentials. The structures were relaxed until the following criteria were met: max_force, 4.5 × 10^–4^ Ha/bohr; rms_force, 3.0 × 10^–4^ Ha/bohr; max_step, 3.0 × 10^–3^ bohr; rms_step, 1.5 × 10^–3^ bohr. For the core/shell QD without surface reconstructions, it was necessary to increase max_force to 2.5 × 10^–3^ Ha/bohr, which could be indicative of strain at the core/shell interface. The isosurface value used for the charge density plots is |0.005| (e^–^/bohr^3^)^1/2^. The inverse participation ratio (IPR) and the crystal orbital overlap population (COOP) (the computational details of which have been discussed previously)^41^ were calculated using the workflows implemented in the nano-QMFlows package.^42^ Further details can be found in the main text and the appendix of ref (42).

## Supporting Information Available

Additional computing details including different methods and software packages, effects of using formate ligands instead of chlorides, limitations observed in the pattern shown in Figure 3D, details on the pattern used for the ⟨100⟩-facets, and modeling features of the CdSe/ZnS core/shell model (PDF)
