# Supplementary material for: Surface Reconstructions in II–VI Quantum Dots
Source: ACS Nano. 2024 Jan 3;18(2):1563–72. doi: 10.1021/acsnano.3c09265 (PMC10795476; doi:10.1021/acsnano.3c09265)
Supplement: Supplementary file 1 — nn3c09265_si_001.pdf [file nn3c09265_si_001.pdf]

Surface Reconstructions in II-VI Quantum Dots

Jordi Llusar[a]*, Indy du Fossé*[b]*, Zeger Hens*[d]*, Arjan Houtepen**[b] *and Ivan Infante**[a,c]

[a]BCMaterials, Basque Center for Materials, Applications, and Nanostructures, UPV/EHU Science Park, Leioa 48940, Spain

[b]Department of Chemical Engineering, Optoelectronic Materials, Delft University of Technology, Van der Maasweg 9, 2629 HZ Delft, The Netherlands

[c]Ikerbasque Basque Foundation for Science, Bilbao 48009, Spain

[d]Physics and Chemistry of Nanostructures, Department of Chemistry, and Center for Nano and Biophotonics, Ghent University, B-9000 Gent, Belgium

Supporting Information

S1. Further details:

(1) On the question if the computational method is wrong

Unless otherwise stated, our calculations in the main text employ a double-ζ basis set and the PBE functional. We also investigated the impact of using a larger triple-ζ basis set, but found no significant effects on the band gap or the shape of the HOMO and LUMO. Furthermore, employing the hybrid PBE0 exchange-correlation functional raises the band gap of Cd_{176} and Cd_{360} to 1.6 and 0.2 eV, respectively, without affecting the surface localization. Moreover, switching to an alternative quantum chemistry software such as ADF[1] instead of CP2K[2] yields no significant changes. Hence, the choice of basis set, functional, or software does not qualitatively impact the results, indicating that the computational method is not an issue here. All these features are illustrated in **Figure S1**.

(2) On the question if the atomistic structure of the modeled QD is wrong

Experimental observations demonstrate that QDs are often coated with a combination of X-type oleate ligands, L-type ligands like amines or phosphines, and Z-type ligands such as $CdCl_2$ or $Cd(RCOO)_2$.[3,4] **Figure S2**, shows that for the large Cd_{360} model, exchanging their chlorides for formate ligands does not have a significant effect on the electronic structure, in line with the conclusion made for Cd_{68} in the main text. Therefore, for computational efficiency, only chloride X-type ligands are typically included to maintain charge balance within the QD.

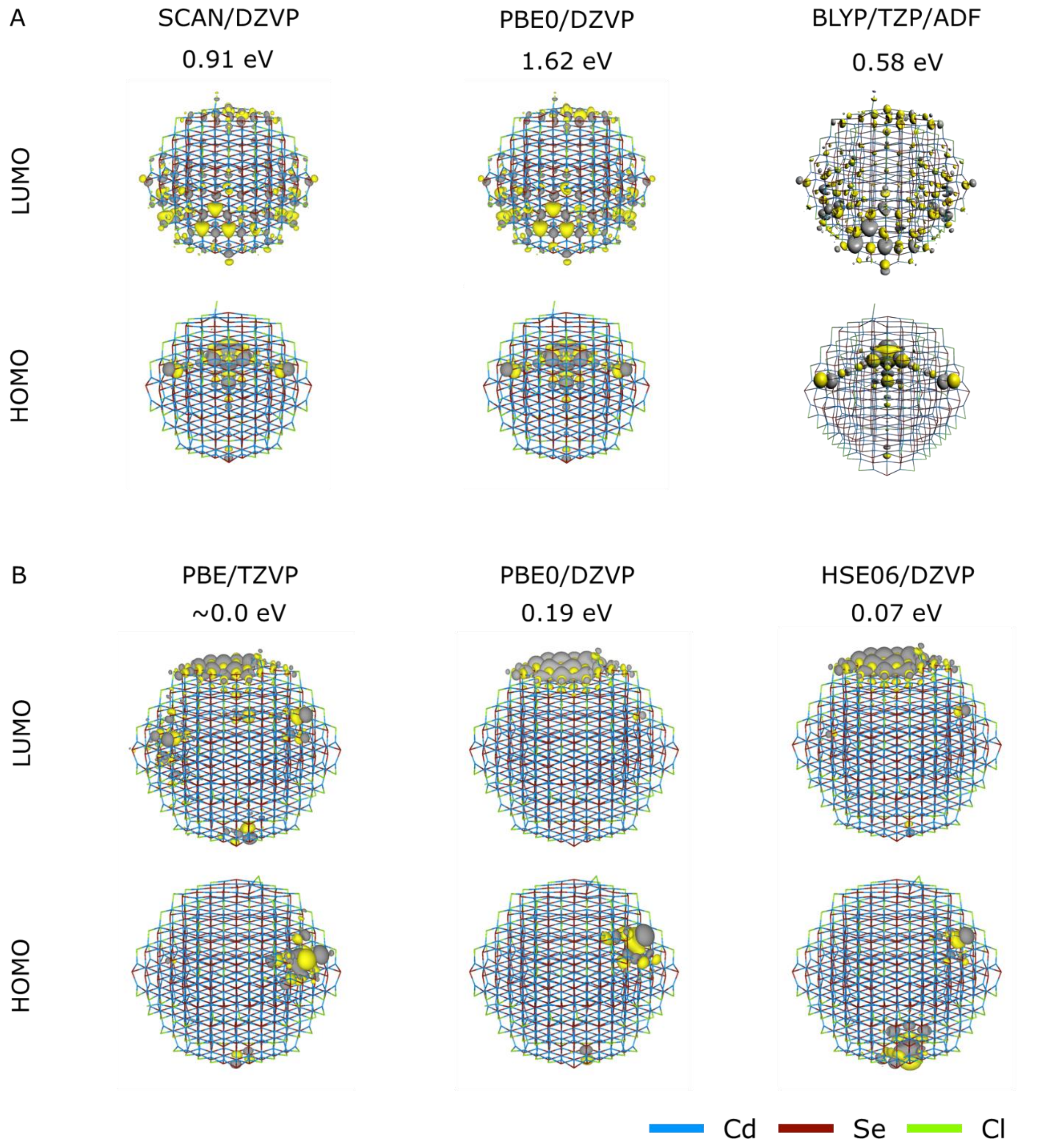

Figure S1. Delocalization and band gap energy computed using different basis sets, functionals, and software packages for the Cd_{176} and Cd_{360} models shown in **Figure 1**. (A) Contour plots of the HOMO and LUMO of Cd_{176} and (B) Cd_{360} systems. The band gap energy value is displayed above each LUMO plot. To reduce computation costs, all calculations are single-point calculations based on the optimized structures presented in **Figure 1**. The CP2K

software package was used for all calculations, except for the BLYP/TZP/ADF calculation, which was performed in ADF.[1]

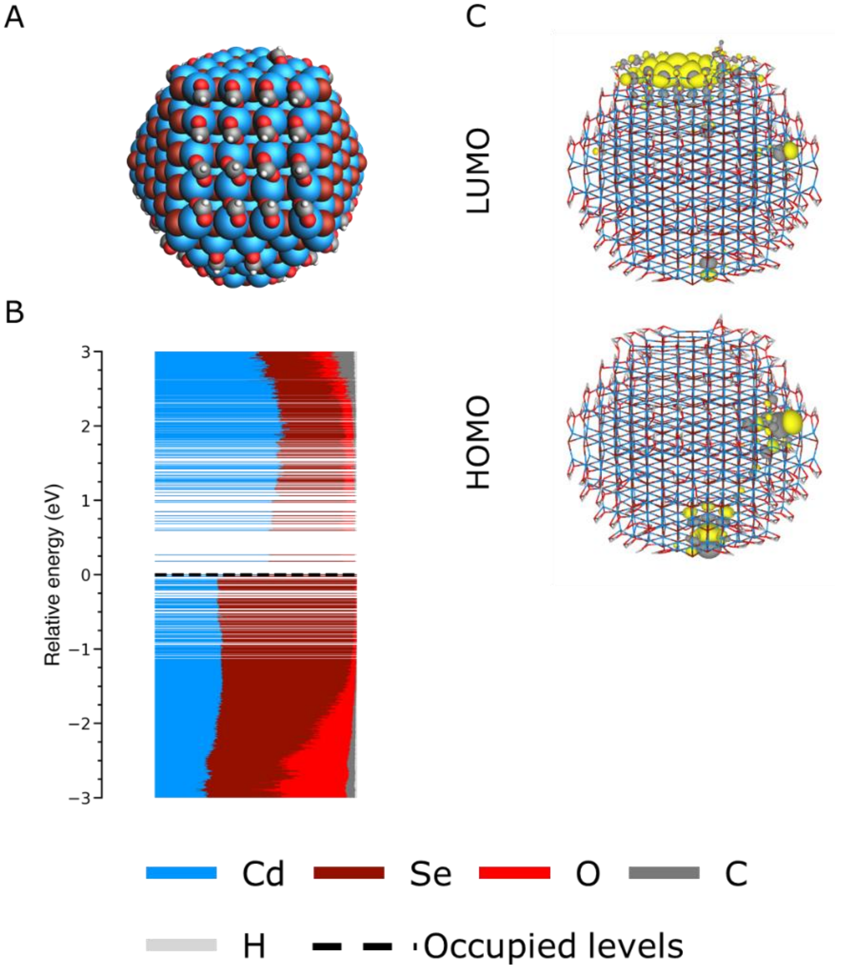

Figure S2. Details of Cd_{360} with formate ligands instead of chlorides. (A) Model, (B) DOS, and (C) contour plots of the HOMO and LUMO.

S2. Nature of the formation of midgap states in binary systems

Introduction

Surfaces break the translational symmetry of the infinite lattice. As shown by the nearly free electron model proposed by Shockley or the tight-binding approach of Tamm,[5,6] surfaces can therefore resul in electronic surface states with an energy within a gap separating two bands of Bloch states. An interesting model to illustrate this point was proposed by Harrison, who considered a finite, one-dimensional (1D) chain of identical atoms that couple through atomic-like orbitals with s and p symmetry.[7] Note that only the p orbital oriented along the atomic chain is considered here. This model enables the formation of surface states to be analyzed analytically, and is readily extended to binary compounds, such as II-VI or III-V semiconductors.

S2.1. General approach for a mono-atomic, 1D sp chain.

Considering the single-electron eigenstates on a finite, 1D chain to be linear combinations of atomic orbitals, eigenstates can be written as:

$$|\psi_i\rangle = \sum_n \left(a_{i,n}|s_n\rangle + b_{i,n}|p_n\rangle\right) \tag{S1}$$

Here, the index n labels the different atoms on the chain, while i refers to the different basis states formed by addition of the s or p orbitals on the atoms. Limiting coupling between adjacent atoms to nearest neighbors, the expansion coefficients are related through equations of the kind:

$$\begin{aligned}(\varepsilon_s - \varepsilon_i)a_{i,n} + V_{ss\sigma}\left(a_{i,n-1} + a_{i,n+1}\right) - V_{sp\sigma}\left(b_{i,n-1} - b_{i,n+1}\right) = 0 \\ \left(\varepsilon_p - \varepsilon_i\right)b_{i,n} + V_{pp\sigma}\left(b_{i,n-1} + b_{i,n+1}\right) + V_{sp\sigma}\left(a_{i,n-1} - b_{i,n+1}\right) = 0\end{aligned} \tag{S2}$$

Following Harrison,[7] we introduce the following Ansatz to make this set of equations independent of the index n:

$$\begin{aligned}a_{i,n} = \alpha_i \phi^n \\ b_{i,n} = \beta_i \phi^n\end{aligned} \tag{S3}$$

Substituting the expansion coefficients $a_{i,k}$ and $b_{i,k}$ in Eqs S2 indeed yields the same set of homogeneous equations for each n:

$$\begin{aligned}&[(\varepsilon_s-\varepsilon_i)+V_{ss\sigma}(\phi^{-1}+\phi)]\alpha_i-V_{sp\sigma}(\phi^{-1}-\phi)\beta_i=0\\&\left[(\varepsilon_p-\varepsilon_i)+V_{pp\sigma}(\phi^{-1}+\phi)\right]\beta_i+V_{sp\sigma}(\phi^{-1}-\phi)\alpha_i=0\end{aligned}\tag{S4}$$

Non-trivial solutions will be obtained if:

$$[(\varepsilon_s-\varepsilon_i)+V_{ss\sigma}(\phi^{-1}+\phi)]\left[(\varepsilon_p-\varepsilon_i)+V_{pp\sigma}(\phi^{-1}+\phi)\right]-V_{sp\sigma}^2(\phi^{-1}-\phi)^2=0\tag{S5}$$

As outlined by Harrison,[7] this relation yields for every energy ε_i four different factors ϕ, which can be labeled as ϕ_1, ϕ_1^{-1}, ϕ_2 and ϕ_2^{-1}. Any factor ϕ that can be written as e^{ikn} will result in a Bloch wave at the corresponding energy. In all other cases, the expansion coefficients will exhibit either an exponential increase or decrease as a function of the atom position n. Hence, only the Bloch waves are acceptable solutions for infinite crystals. For finite crystals, however, this is not necessarily the case.

S2.2. Surface-states in a semi-infinite mono-atomic, 1D *sp* chain.

Focusing on a semi-infinite mono-atomic chain starting with atom $n=1$, the boundary conditions for eigenstates are that the expansion coefficients on both the *s* and *p* states vanish at $n=0$, and that expansion coefficients do not show an exponential increase for $n\to\infty$. These conditions can be met even in the case where neither ϕ_1 nor ϕ_2 would yield a Bloch wave in the bulk crystal. Indeed, taking ϕ_1 and ϕ_2 as the expansion coefficients that decay exponentially for $n\to\infty$, one can consider the state $|S\rangle=c_1|\phi_1\rangle+c_2|\phi_2\rangle$. This state will solve the equations for the expansion coefficients and fit the boundary conditions, provided that the expansion coefficients of $|S\rangle$ on the s and p orbital at $n=0$ vanish, i.e.:

$$\begin{aligned}&a_{1,0}c_1+a_{2,0}c_2=0\\&b_{1,0}c_1+b_{2,0}c_2=0\end{aligned}\tag{S6}$$

Using the Ansatz for the expansion coefficients, this condition can be rewritten as:

$$\alpha_1 c_1 + \alpha_2 c_2 = 0$$
$$\beta_1 c_1 + \beta_2 c_2 = 0 \qquad \text{(S7)}$$

Hence, the factors ϕ_1 and ϕ_2 that solve Eq S5 for a given energy will give an eigenstate proper at that energy provided that the corresponding coefficients α and β have the following property:

$$\frac{\alpha_1}{\beta_1} = \frac{\alpha_2}{\beta_2} \qquad \text{(S8)}$$

Using Eqs S4 that related the different expansion coefficients, one demonstrates that this relation can be met provided that the band-gap features an inversion, meaning that the bulk *s*-band and bulk *p*-band cross if the *sp* coupling would be turned off. Under such a condition, a surface state is obtained at the energy ε_S of:[7]

$$\varepsilon_S = \frac{V_{pp\sigma}\varepsilon_s - V_{ss\sigma}\varepsilon_p}{V_{pp\sigma} - V_{ss\sigma}} \qquad \text{(S9)}$$

S2.3. Numerical example

To illustrate Harrison's theory, we numerically solved the Schroedinger equation for a mono-atomic 1D chain of 32 atoms with *sp* coupling, where by choice of the on-site energies and the coupling matrix elements, *sp* crossing can be turned on or off. **Figure S3** shows a first example for a chain without sp crossing.

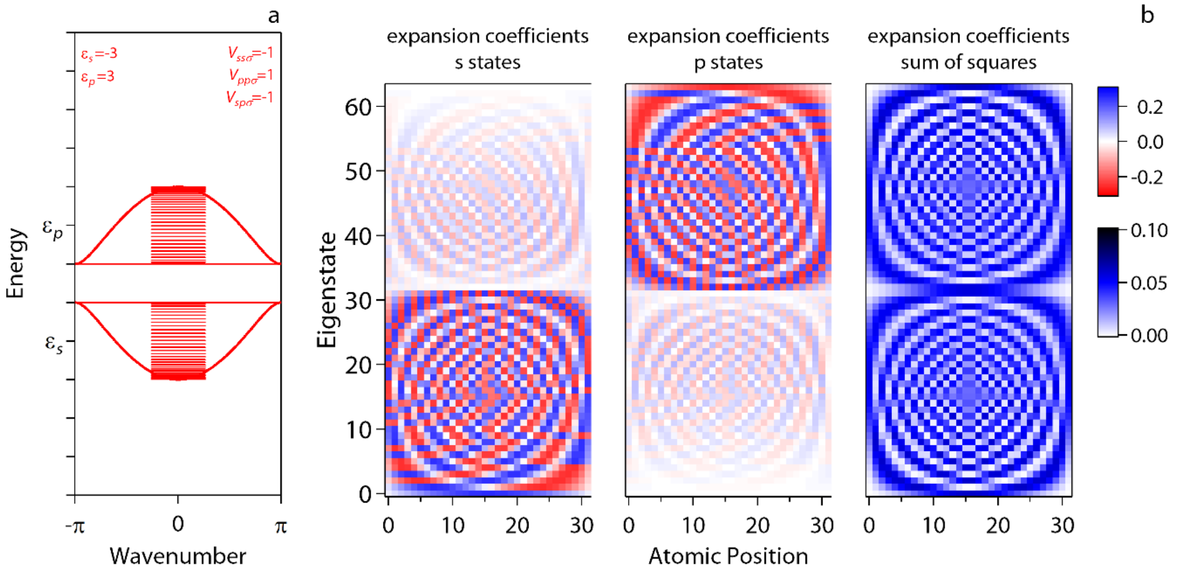

Figure S3. Electronic states on a mono-atomic 1D chain with *sp* coupling without inversion of the band edges. (a) Representation of (full line) the bulk dispersion relation and (horizontal lines) the discrete states as obtained for a 32 atom chain. All states of the finite chain fall within the allowed bands of bulk energy levels. (b) Representation of the expansion coefficients of the different eigenstates for (left) the *s* orbitals and (middle) the *p* orbitals together with (right) the total sum of squares of the expansion coefficients on each atom. In line with the parameter settings, states 31 and 32 at the upper edge of the valence band and the lower edge of the conduction band consist of contributions from s orbitals and p orbitals respectively, i.e., no inversion of the bands. On-site energies and coupling matrix elements were taken as indicated.

Figure S3a displays the energy of the 64 eigenstates on top of the bulk dispersion, while **Figure S3b** provides the expansion coefficients of each of these eigenstates for the s and p orbital on each atom, together with the sum of squares of these expansion coefficients on each atom. Here, the horizontal axis labels the atom position, while the different eigenstates are separated along the vertical axis in order of increasing energy. Note that the lowest energy state is labeled as 0.

As can be seen in **Figure S3a**, the energy of all states fall within the range of allowed energy bands of the corresponding bulk material. Moreover, as expected in the absence of inversion, the uppermost valence-band state (state 31) consists of an anti-bonding combination of *s* orbitals,

and the lowermost conduction-band state (state 32) of a bonding combination of *p* orbitals that are modulated by a sine-like envelope that has nodes just beyond the edges of the crystal. Moving down into the valence band or up into the conduction band, one sees that the envelope develops additional nodes with the chain, as expected for quantized states on a finite chain.

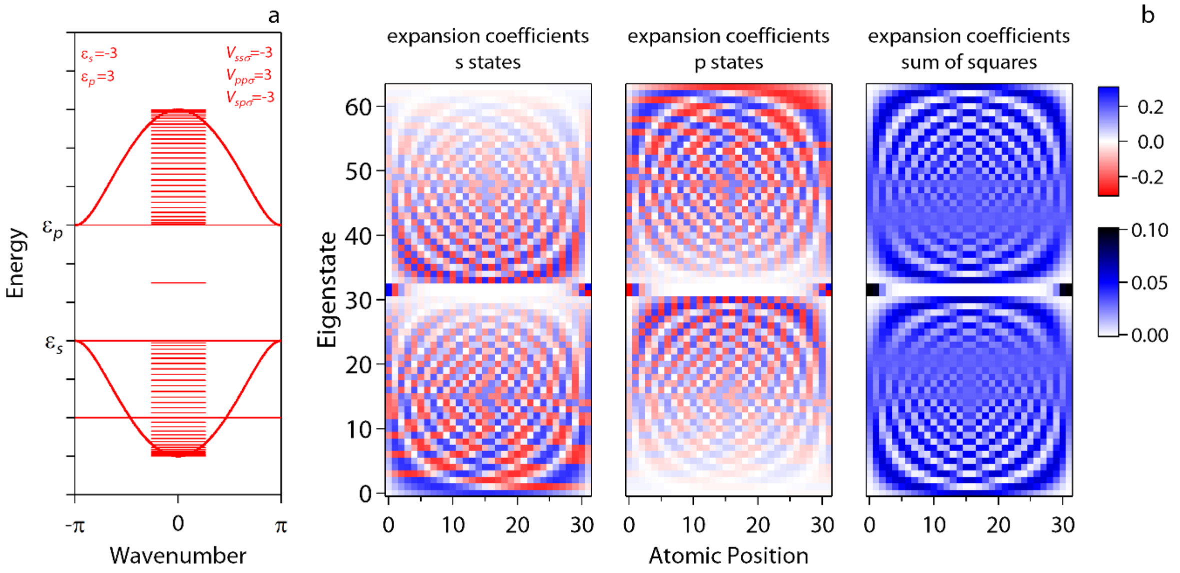

Figure S4. Electronic states on a mono-atomic 1D chain with *sp* coupling with inversion of the band edges. (a) Representation of (full line) the bulk dispersion relation and (horizontal lines) the discrete states as obtained for a 32 atom chain. Note the appearance of an energy level within the gap separating the two bands of allowed bulk states at the position predicted by Eq S10. (b) Representation of the expansion coefficients of the different eigenstates for (left) the *s* orbitals and (middle) the *p* orbitals together with (right) the total sum of squares of the expansion coefficients on each atom. In line with the parameter settings, states 30 and 33 at the upper edge of the valence band and the lower edge of the conduction band consist of contributions from p orbitals and s orbitals respectively, i.e., inversion of the bands. This band inversion comes with the formation of two surface-localized states. On-site energies and coupling matrix elements were taken as indicated.

Figure S4 represents the results of a similar calculation, but for a parameter setting that leads to an inversion of the bands. In this case, the state at the top of the valence band (state 30) is a bonding combination of *p*-states while that at the bottom of the conduction band (state 33) is an anti-

bonding combination of *s*-states; a change in order that reflects the band inversion. Moreover, a two-fold degenerate surface state (state 31 and 32) appears for this parameter setting. While the numerical analysis yields states in agreement with the inversion symmetry of the chain, considering the sum and the difference of these states yields one surface state localized at the left edge of the chain and one localized at the right edge. Interestingly, at the left, the expansion coefficients for the *s* and *p* orbital on the atom at the left edge have opposite sign, while for the atom at the right edge, they have the same sign. These combinations can be seen as leading to hybrid *sp* orbitals pointing away from the chain at either edge, i.e., a dangling bond.

S2.4. Extension to diatomic chains

The approach proposed by Harrison can be readily extended to diatomic chains consisting of a more electronegative element – the anion A – and a less electronegative element – the cation B – that couple by means of *s* and *p* orbitals. Again, depending on the choice of the on-site and coupling energies, an inversion of the bands can be turned on or off. Note that II-VI and III-V semiconductors, such as CdSe and InP, typically show inverted bands with the top of the valence band consisting of anion p orbitals and the bottom of the conduction band of cation s orbitals.[8] To illustrate the impact of band inversion, we solved numerically the Schroedinger equation for a chain consisting of 32 unit cells, i.e., 64 atoms in total, starting with an anion at its left, and ending with a cation at its right.

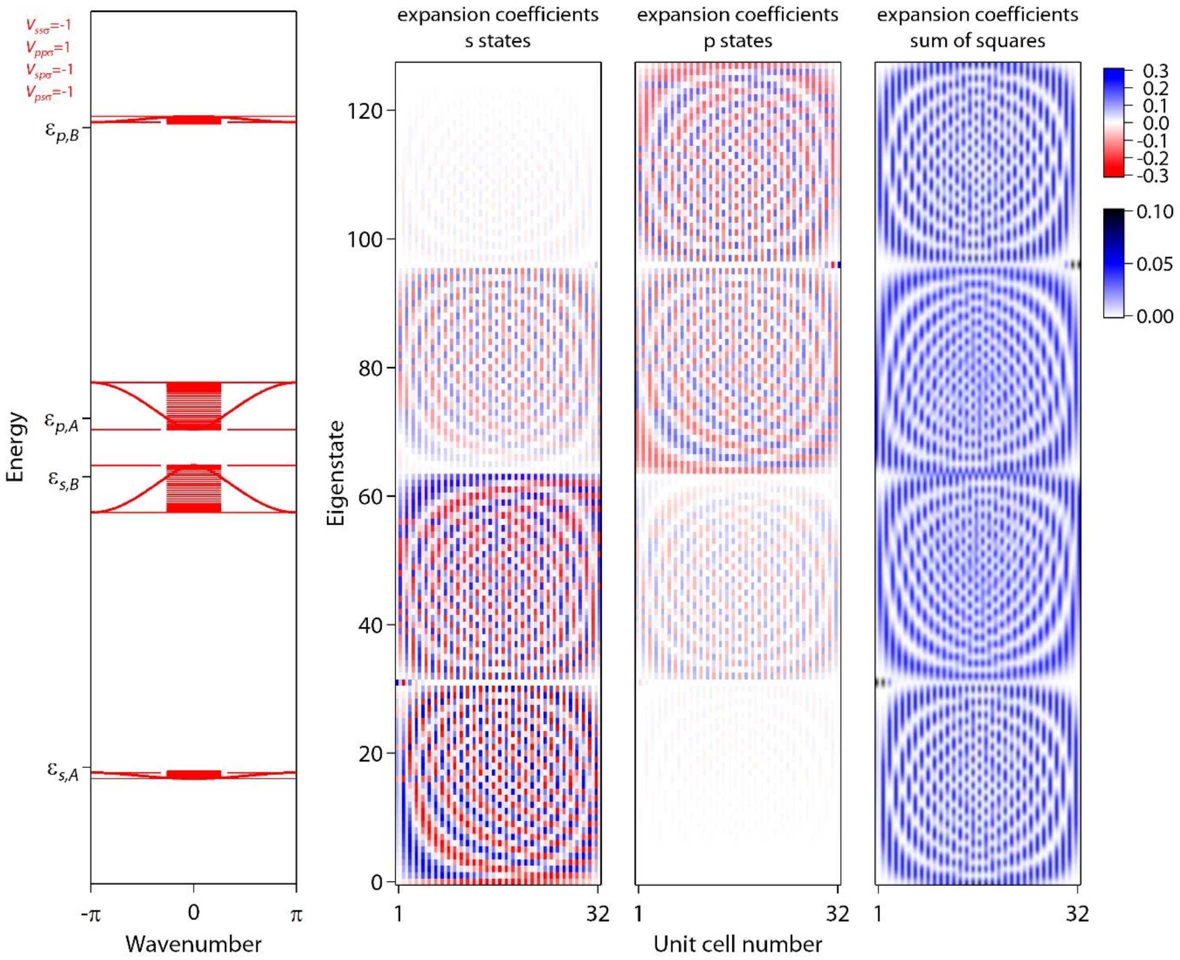

Figure S5. Electronic states on a binary 1D chain with *sp* coupling without inversion of the band edges. (a) Representation of (full line) the bulk dispersion relation and (horizontal lines) the discrete states as obtained for a chain consisting of 32 unit cells. (b) Representation of the expansion coefficients of the different eigenstates for (left) the *s* orbitals and (middle) the *p* orbitals together with (right) the total sum of squares of the expansion coefficients on each atom. The horizontal axis labels successive unit cells, where the anion is at the left and the cation at the right side of each unit cell. In line with the parameter settings, states 31 and 32 at the upper edge of the valence band and the lower edge of the conduction band consist of contributions from s orbitals and p orbitals respectively, i.e., no inversion of the bands. On-site and coupling energies were taken as indicated.

Figure S5 represents the eigenenergies and eigenstates in a similar way as **Figure S3** and **S4** for the monoatomic chain. Note, however, that the horizontal axis in **Figure S5b** labels the unit cells, with two per unit cell. Moreover, having 4 atomic states per unit cell, we obtain 4 energy bands

which we will indicate as the lower and upper valence band and the lower and upper conduction band, respectively. As can be seen, all states labeled between 32 and 95 fall within the ranges of the upper valence band or the lower conduction band of the corresponding bulk chain. Moreover, the uppermost valence-band state (state 63) is a fully anti-bonding combination of A and B s orbitals, while the lowermost conduction band state (state 64) is a fully bonding combination of A and B p orbitals. No surface states appear in between these states. Interestingly, surface states do appear just above the lower valence band and just below the upper conduction band. However, such states will have no impact on the opto-electronic properties of the chain.

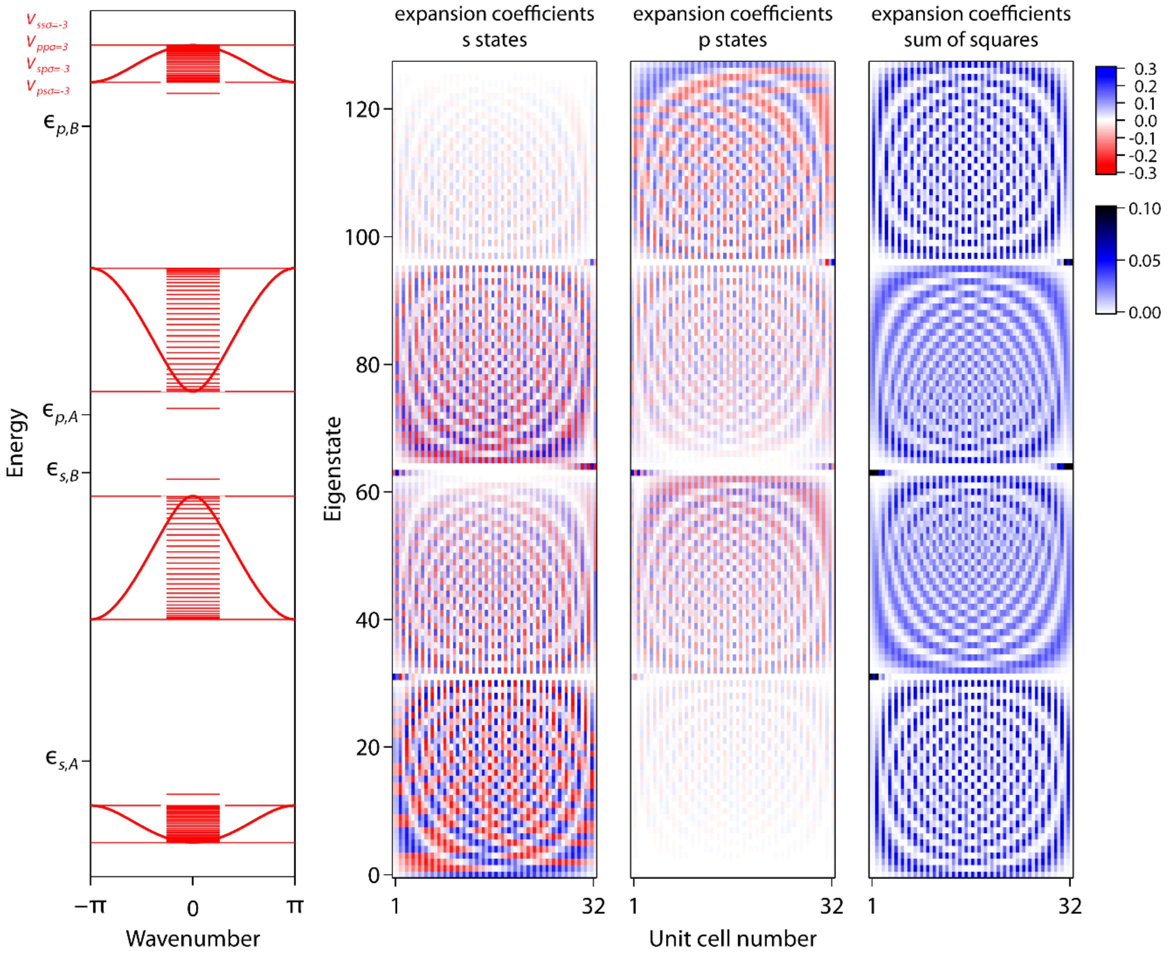

Figure S6. Electronic states on a binary 1D chain with *sp* coupling with inversion of the band edges. (a) Representation of (full line) the bulk dispersion relation and (horizontal lines) the discrete states as obtained for a chain consisting of 32 unit cells. (b) Representation of the expansion coefficients of the different eigenstates for (left) the *s* orbitals and (middle) the *p* orbitals together with (right) the total sum of squares of the expansion coefficients on each atom. The horizontal axis labels successive unit cells, where the anion is at the left and the cation at the right side of each unit cell. In line with the parameter settings, states 30 and 33 at the upper edge of the valence band and the lower edge of the conduction band consist of contributions from p orbitals and s orbitals respectively, i.e., an inversion of the bands. Two surface states, one centered on the anion at the left edge, one on the cation at the right edge of the chain appear within the energy gap separating the upper valence band and the lower conduction band. On-site and coupling energies were taken as indicated.

between both edge states, two surface states appear, a first localized on the anion at the left edge of the chain and a second on the cation at the right edge of the chain. We thus conclude that for binary AB crystals that have an inverted band-gap, the appearance of surface-localized states in finite crystals should be seen as an intrinsic feature, and not an artefact of the calculation.

We highlight that the surface states arising here, are fundamentally different from the states related to the presence of 2-coordinated selenium at the surface of CdSe quantum dots.[9] These states mostly coincide with a Se *p* state that is perpendicular to the plane formed by the Se atom and the two remaining nearest-neighbor Cd atoms; a configuration for which that Se *p* state does not couple with Cd *s* states. Here, surface states arise on a chain where the coupling of the orbitals of the edge atoms is not undone by symmetry, but rather because a finite chain can support an additional class of eigenstates apart from those derived from Bloch waves. The result are two surface states, one formed on the outermost anion and one on the outermost cation that rather resemble an *sp* hybrid pointing away from the chain rather than uncoupled *p* orbitals perpendicular to the chain.

S2.5. Extension to finite two dimensional diatomic crystals

Moving from a 1D chain to 2D crystals, a larger diversity of surface states will arise. Considering the Ansatz S3, an infinite 2D crystal will, once again, only support Bloch states. However, other solutions may feature a combination of a Bloch part in one direction, and an exponential change of the expansion coefficients in a second direction, or two exponentials in two directions. In finite 2D crystals, these combinations can give rise to eigenstates proper, localized on a crystal edge – with the Bloch part parallel to the edge – or in crystal corners.

To illustrate this point, and connect the results obtained on a 1D chain with the outcome of the DFT study on the electronic structure of semiconductor nanocrystals presented in the main text, we applied a similar tight binding approach to the eigenstates of squares cut from an AB honeycomb lattice. In line with the 1D chain, we focus here on the states formed within the sp^2 bands, which are perpendicular to the p system that results from the coupling between adjacent p_z orbitals and are more representative of the states formed within the sp^3 bands of a tetragonal crystal. More specifically, we determined the eigenstates for a square-like nanocrystal, consisting of 12 hexagonal cells in the x direction and 8 in the y direction for a total of 400 atoms – 200 cations and 200 anions – and thus 1200 eigenstates, which we label as 1 to 1200 with increasing energy. Note that the crystal is cut with the anions on top and the cations at the bottom, which makes that the crystal has a mirror symmetry along the y-axis.

Figure S7a represents the energy of the subset of eigenstates that fall between the bottom of the valence band and the top of the conduction band together with the energy bands of the infinite 2D crystal. As can be seen, the majority of the states fall within the range of allowed Bloch state energies, and a small set of discrete states – 627 and higher, 597 and lower – can be distinguished close to the edge of the conduction and valence band, respectively. In addition, a band of states – from state 603 to 626 – is present relatively close to the conduction-band edge, but within the

forbidden gap for Bloch states. This band is separated from a pair of states – 601 and 602 – deeper in this gap, while two more states – 599 and 600 – have an energy slightly higher than the valence-band edge. Note that state 600 – which will be the last occupied level – falls within this forbidden gap.

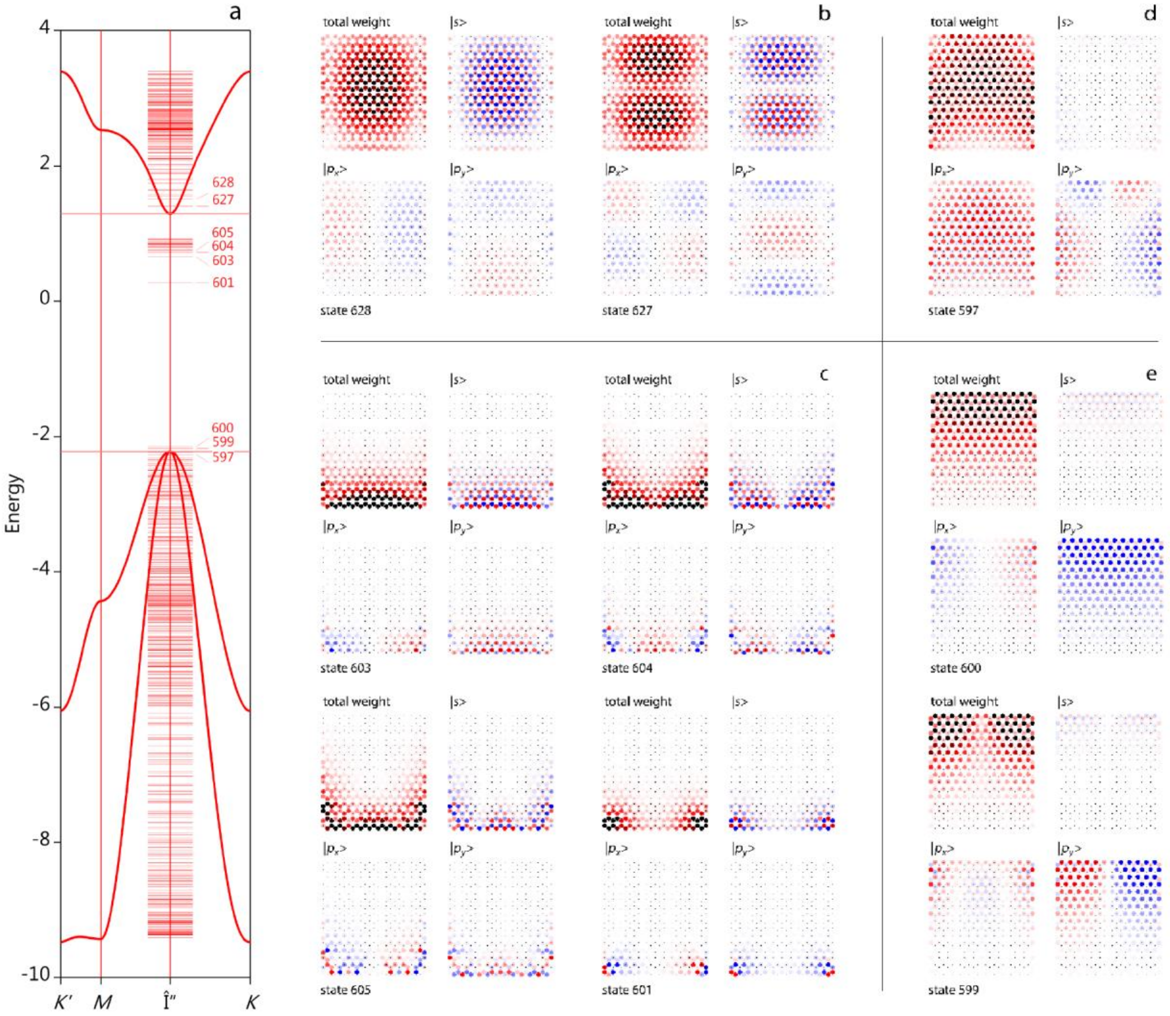

Figure S7. Electronic states on a finite, binary 2D honeycomb with sp^2 bands featuring inversion of the band edges. (a) Representation of (full line) the bulk dispersion relation and (horizontal lines) the discrete states as obtained for a square-like lattice 12 by 8 hexagons in size. (b-e) Representation of (top left) sum of squares of all expansion coefficients per atom, (top right) expansion coefficients of the *s* orbitals, (bottom left) expansion coefficients of the p_x orbitals, and (bottom right) expansion coefficients of the p_y orbitals for the eigenstates as

indicted. Expansion coefficients are printed as color coded spheres at each atomic location, with red positive and blue negative values. The same color coding is used for all the figures. For p_x and p_y orbitals, a positive expansion coefficient represents the orbital with its positive lobe in the positive x (right) and positive y (up) direction, respectively. Parameter setting as in **Figure S6**, with $V_{pp\text{p}}$=-2 as additional coupling energy.

Figure S7b displays the characteristics of the first two discrete states within the conduction band. As expected, these states feature the delocalized standing Bloch-waves composed of anti-bonding s states, in agreement with simplified size quantization models. Note that the even parity of both states forces the expansion coefficients of the p_x states to change sign at the left and right side of the crystal, given the uneven parity of these states with respect to the vertical axis.

Figure S7c displays the characteristics of the three lowest states (603-605) within the first band of mid-gap states. Clearly, these states are still dominated by the cation s states, but they are only delocalized along the bottom edge of the crystal and decay exponentially along the vertical axis. What distinguishes the states along the edge, is the different envelope with (603) no, (604) one or (605) two nodes along the edge. State 601, on the other hand, does not delocalize along the edge but is localized within the corners of the crystal. Coming in a pair with state 602, their sum and difference yields genuinely corner-localized states.

A similar picture, albeit less pronounced, emerges at the valence-band edge. State 597 is again a delocalized state, shifted away from the valence-band edge by size quantization. Opposite from the CB states, the underlying Bloch wave consist of a bonding combination of p orbitals. Note again the different impact of parity on the expansion coefficients of the p_x and p_y orbitals. The near-edge states within the forbidden gap, are now localized on the anion edge, while featuring a slow but steady decay towards the cation edge. Again, the difference between the two states shown is the additional node in the envelope parallel to the edge for state 598.

Note that the exact appearance of these states within the forbidden gap, and the rapid or slow decay of the expansion coefficients within the bulk of the crystal, will depend on the parameter settings, and thus the material under investigation. The example shown here merely serves to illustrate that a band of edge and corner localized states are to be expected in 2D nanocrystals with edges merely cut from a bulk crystal. By extension, one can expect surface, edge and corner localized states appearing in 3D nanocrystals.

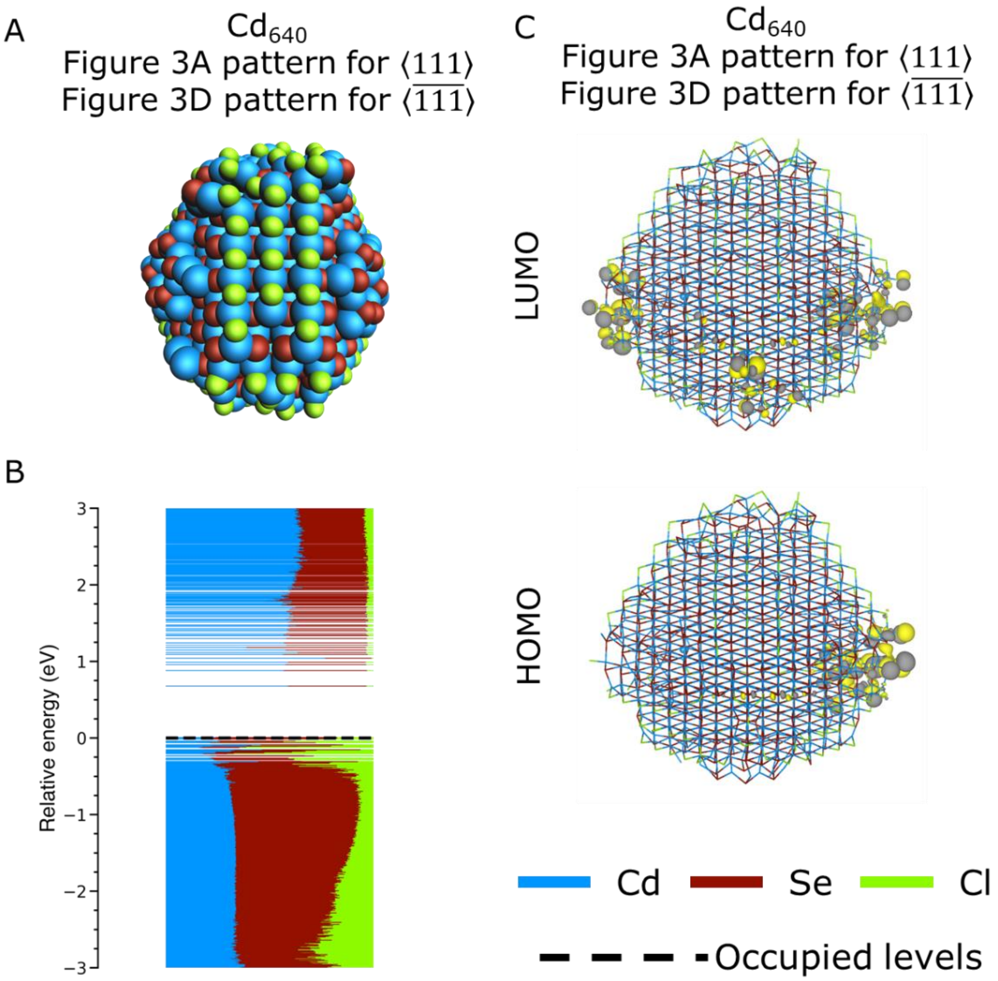

Figure S8. Details of Cd_{640} with amine and $CdCl_2$ ligands, and Cd_{640} with the surface reconstruction pattern as shown in **Figure 3A** for ⟨111⟩-facets and **Figure 3D** for ⟨$\overline{111}$⟩-facets. (A) Structures of the two QDs. (B) DOS and (C) contour plots of the HOMO and LUMO of each system.

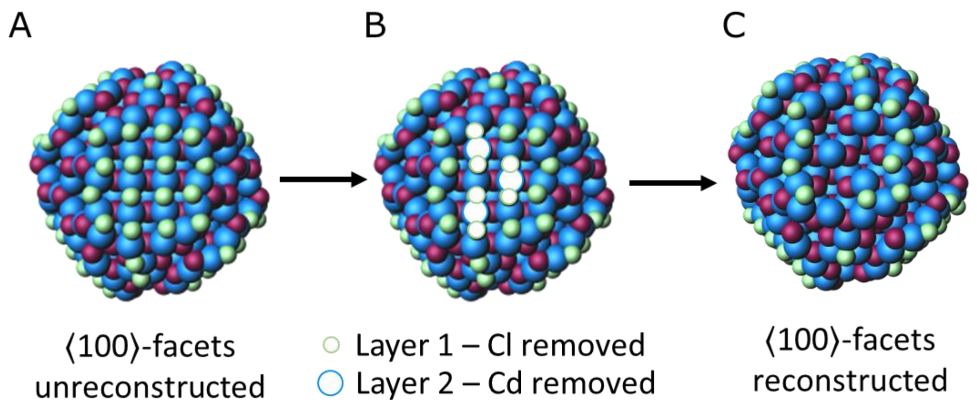

Figure S9. Cd_{360} model sketch: (A) with ⟨100⟩-facets unreconstructed, (B) showing the removal of the Cd and Cl atoms, and (C) with ⟨100⟩-facets reconstructed.[10]

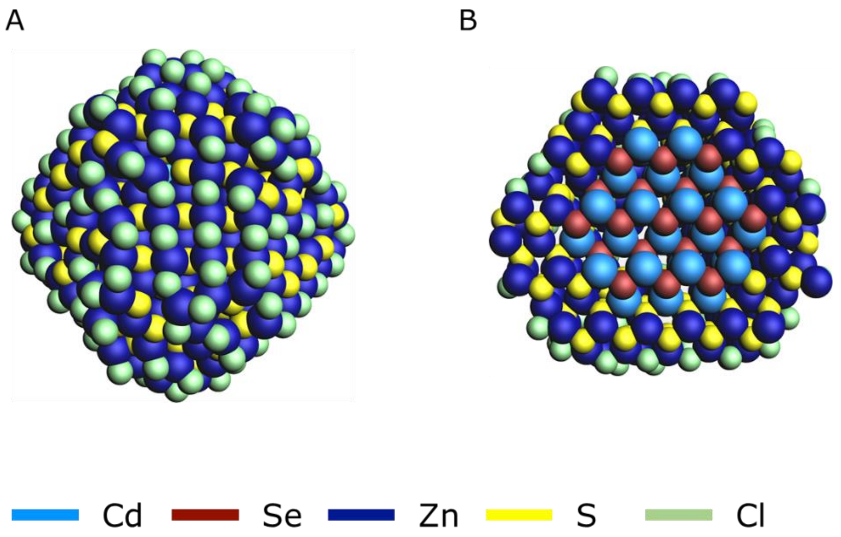

Figure S10. Structure of the core/shell QD model shown in **Figure 5**. (A) External view of the $Cd_{68}Se_{55}Zn_{292}S_{226}Cl_{158}$ model. (B) Sliced view of the QD, showing the $Cd_{68}Se_{55}$ core.

S3. PDOS for Core/Shell systems

A useful tool for analyzing the molecular orbital contributions that different atomic elements have on the energy levels in a quantum dot is the partial density-of-states (PDOS) plot. To

understand how much a given atomic element contributes to one-electron levels at certain energies, we use the following expression:

$$PDOS_A(E) = \sum_i C_{A,i} F(E - \varepsilon_i) \tag{S10}$$

where $C_{A,i}$ represents the orbital contribution i for each atom type A, and $F(E - \varepsilon_i)$ denotes a Gaussian function.

Although equation (S10) works well for systems containing only one domain (e.g., core-only quantum dots), it might present some problems in core-shell structures, when the number of atoms in the core is much smaller than the number of atoms in the shell. This is a common occurrence in QDs and a regular DOS might show a predominant shell character even though the contribution of each shell atom is very small. To address this issue, we introduce a new renormalization of the PDOS and to so we introduce a new term:

$$PDOS'_{k,i}(E) = \frac{1}{N_k} \sum_{k=1}^{N_k} C_{k,i} \quad ; \quad k = core, shell \tag{S12}$$

$PDOS'_k(E)$ differs from equation (S10) by a factor of $\frac{1}{N_k}$, i.e. we consider the average contribution of each atom in the core. Furthermore, we consider for simplicity $F(E - \varepsilon_i) = 1$, although the expression will be still valid in general. In (S12), N_k represents the number of atoms in the domain k (core or shell) and the sum is over all the atoms within a given domain k. Since equation (S12) is not yet renormalized, a renormalization factor is required. Let us particularize equation (S12) for the domains k, core and shell:

$$\mathbf{N} \left(PDOS'_{core,i}(E) + PDOS'_{shell,i}(E) \right) = 1 \tag{S13}$$

Taking equation (S12) into equation (S13) and solving for **N**, we find:

$$\mathbf{N} = \frac{1}{\left(\frac{1}{N_{core}} \sum_{core=1}^{N_{core}} C_{core,i} + \frac{1}{N_{shell}} \sum_{shell=1}^{N_{shell}} C_{shell,i} \right)} \tag{S14}$$

Now, the denominator in equation (S14) can be seen as the sum of $PDOS'_k(E)$ for the core and shell domains and then, can be rewritten as:

$$\mathbf{N} = \frac{1}{TDOS'_{core-shell,i}(E)} \tag{S15}$$

Therefore, plugging equation (S15) into equation (S14), we obtain the following expression:

$$\frac{PDOS'_{core,i}(E)}{TDOS'_{core-shell}(E)} + \frac{PDOS'_{shell,i}(E)}{TDOS'_{core-shell,i}(E)} = 1 \quad \text{(S16)}$$

We then define a normalized PDOS for each domain as:

$$NPDOS'_{core,i}(E) = \frac{PDOS'_{core,i}(E)}{TDOS'_{core-shell}(E)}$$

$$NPDOS'_{shell,i}(E) = \frac{PDOS'_{shell,i}(E)}{TDOS'_{core-shell}(E)}$$

In this way we obtain a more balanced description for the contribution of the core and shell.

(1) te Velde, G.; Bickelhaupt, F. M.; Baerends, E. J.; Fonseca Guerra, C.; van Gisbergen, S. J. A.; Snijders, J. G.; Ziegler, T. Chemistry with ADF. *J. Comput. Chem.* **2001**, *22*, 931–967.

(2) Hutter, J.; Iannuzzi, M.; Schiffmann, F.; Vandevondele, J. CP2K: Atomistic Simulations of Condensed Matter Systems. *Wiley Interdiscip. Rev. Comput. Mol. Sci.* **2014**, *4*, 15–25.

(3) Anderson, N. C.; Hendricks, M. P.; Choi, J. J.; Owen, J. S. Ligand Exchange and the Stoichiometry of Metal Chalcogenide Nanocrystals: Spectroscopic Observation of Facile Metal-Carboxylate Displacement and Binding. *J. Am. Chem. Soc.* **2013**, *135*, 18536–18548.

(4) Boles, M. A.; Ling, D.; Hyeon, T.; Talapin, D. V. The Surface Science of Nanocrystals. *Nat. Mater.* **2016**, *15*, 141–153.

(5) Shockley, W. On the Surface States Associated with a Periodic Potential. *Phys. Rev.* **1939**, *56* (4), 317–323.

(6) Tamm, I. On the Possible Bound States of Electrons on a Crystal Surface. *Phys. Z. Sowjetunion* **1932**, *1*, 733–735.

(7) Harrison, W. Tight-Binding Theory of Surface States in Metals. *Phys. Scr* **2003**, *67* (3), 253–259.

(8) Yakovkin, I. N.; Petrova, N. V. Band Inversion and Absence of Surface States in IV – VI Semiconductors. *Physics Letters, Section A: General, Atomic and Solid State Physics* **2021**, *403*.

(9) Houtepen, A. J.; Hens, Z.; Owen, J. S.; Infante, I. On the Origin of Surface Traps in Colloidal II-VI Semiconductor Nanocrystals. *Chem. Mater.* **2017**, *29*, 752–761.

(10) Tatarenko, S.; Bassani, F.; Klein, J. C.; Saminadayar, K.; Cibert, J.; Etgens, V. H. Surface Reconstructions of (001) CdTe and Their Role in the Dynamics of

Evaporation and Molecular-beam Epitaxy Growth. *J. Vac. Sci. Technol. A: Vacuum, Surfaces, and Films* **1994**, *12*, 140–147.
